# Supplementary material for: Lead-free dual-frequency ultrasound implants for wireless, biphasic deep brain stimulation
Source: Nat Commun. 2024 May 13;15:4017. doi: 10.1038/s41467-024-48250-z (PMC11091107; doi:10.1038/s41467-024-48250-z)
Supplement: Supplementary file 1 — Supplementary Information [file 41467_2024_48250_MOESM1_ESM.pdf]

Supplementary Information for

Lead-free dual-frequency ultrasound implants for wireless, biphasic deep brain stimulation

Qian Wang[1, †], Yusheng Zhang[2, †], Haoyue Xue[1], Yushun Zeng[3], Gengxi Lu[3], Hongsong Fan[2,*], Laiming Jiang[1,*], Jiagang Wu[1,*]

[1]College of Materials Science and Engineering, Sichuan University, Chengdu, 610064, China.

[2]National Engineering Research Center for Biomaterials, Sichuan University, Chengdu 610064, China

[3]Alfred E. Mann Department of Biomedical Engineering, Viterbi School of Engineering, University of Southern California, Los Angeles, California 90089, USA.

[*] Corresponding author. Email: hsfan@scu.edu.cn (H. Fan), laimingjiang@scu.edu.cn (L. Jiang), wujiagang0208@163.com (J.W.)

[†] Q. Wang and Y. Zhang contributed equally to this work.

Contents

Supplementary Figures

Supplementary Fig. 1 | Design and advantages of the external dual-transducer with soft connection.

Supplementary Fig. 2 | Comparison of piezoelectric output in the focused and plane-wave acoustic fields when the harvester position is varied.

Supplementary Fig. 3 | Surface SEM images of ceramics.

Supplementary Fig. 4 | Phase fraction of each phase of KNNS95 ceramics.

Supplementary Fig. 5 | Optical image of the porous ceramic cross-section.

Supplementary Fig. 6 | Room temperature XRD patterns and magnified (200)pc diffraction peaks of sandwich ceramics with varying diameters.

Supplementary Fig. 7 | Temperature-dependent dielectric constants of sandwich ceramics with varying diameters.

Supplementary Fig. 8 | Domain structural characterization of the ceramics measured by PFM.

Supplementary Fig. 9 | Electrical properties of ceramics with different conditions.

Supplementary Fig. 10 | Polarization-electric field (P-E) hysteresis loops of D-C, S-C, and SP-1-3 composites.

Supplementary Fig. 11 | Ferroelectric properties of the sandwich ceramics with varied proportion of PS.

Supplementary Fig. 12 | Strain properties of the sandwich ceramics with varied proportion of PS.

Supplementary Fig. 13 | Ferroelectric properties of the sandwich ceramics with varied diameters of PS.

Supplementary Fig. 14 | Strain properties of the sandwich ceramics with varied diameters of PS.

Supplementary Fig. 15 | Schematic of the measurement setup for ultrasound energy transfer system.

Supplementary Fig. 16 | Comparison between the trigger signals and output signal in PUEH-2.

Supplementary Fig. 17 |. Performance characterization of the PUEH-1.

Supplementary Fig. 18 | Output voltages under the excitation of trigger signals with different cycles.

Supplementary Fig. 19 | Output voltages of the sample measured in a continuous mode.

Supplementary Fig. 20 | Characterization of the ultrasound-induced electrical outputs of f-BUI at 1 MHz channel in the ex vivo porcine experiment.

Supplementary Fig. 21 | Relationship between the output of the harvester and the incidence angle of the ultrasound wave.
Supplementary Fig. 22 | Impedance spectra of two S-1-3 PUEHs with different resonance frequencies (1 MHz and 3 MHz).
Supplementary Fig. 23 | Output performance comparison of two harvesters with different frequencies.
Supplementary Fig. 24 | Dual-alternating current (AC) trigger waveforms.
Supplementary Fig. 25 | Biphasic stimulus pulse generated by the f-BUI, where the amplitudes of biphasic stimulation waveform can be modulated by adjusting the trigger voltages of both channels simultaneously.
Supplementary Fig. 26 | Biphasic stimulus pulse generated by the f-BUI, where the amplitudes of biphasic stimulation waveforms is significantly different under resonant and non-resonant operation.
Supplementary Fig. 27 | Implantation procedure for the f-BUI for rats.
Supplementary Fig. 28 | Micro-CT imaging and photographs of f-BUI in the rat brain.
Supplementary Fig. 29 | Front view and top view of skull phantom with the top f-BUI device.
Supplementary Fig. 30 | ECoG signals record in Sham group treated by US+f-BUI.
Supplementary Fig. 31 | Quantification of the cells number in the CA3 region after different treatment.
Supplementary Fig. 32 | Comparison of electrolysis experiments by using monophasic pulses and biphasic pulses.
Supplementary Fig. 33 | Comparison of stimulation experiments in vivo by using monophasic pulses and biphasic pulses.

Supplementary Tables

Supplementary Tab. 1 | Comparison of device dimensions for brain implantation in rodent models.
Supplementary Tab. 2 | Refinement results of KNNS95.
Supplementary Tab. 3 | Comparison of d_{33}, ε_r, g_{33} and $d_{33} \times g_{33}$ values between D-C, S-C and SP-1-3 composites.
Supplementary Tab. 4 | Comparison of the transduction performance of f-BUI and other representative energy harvesters.

Supplementary Notes

Supplementary Note 1 | Preparation of KNNS95 ceramics (D-C and S-C).
Supplementary Note 2 | Fabrication of dual transducer.

Supplementary References

Supplementary Figures

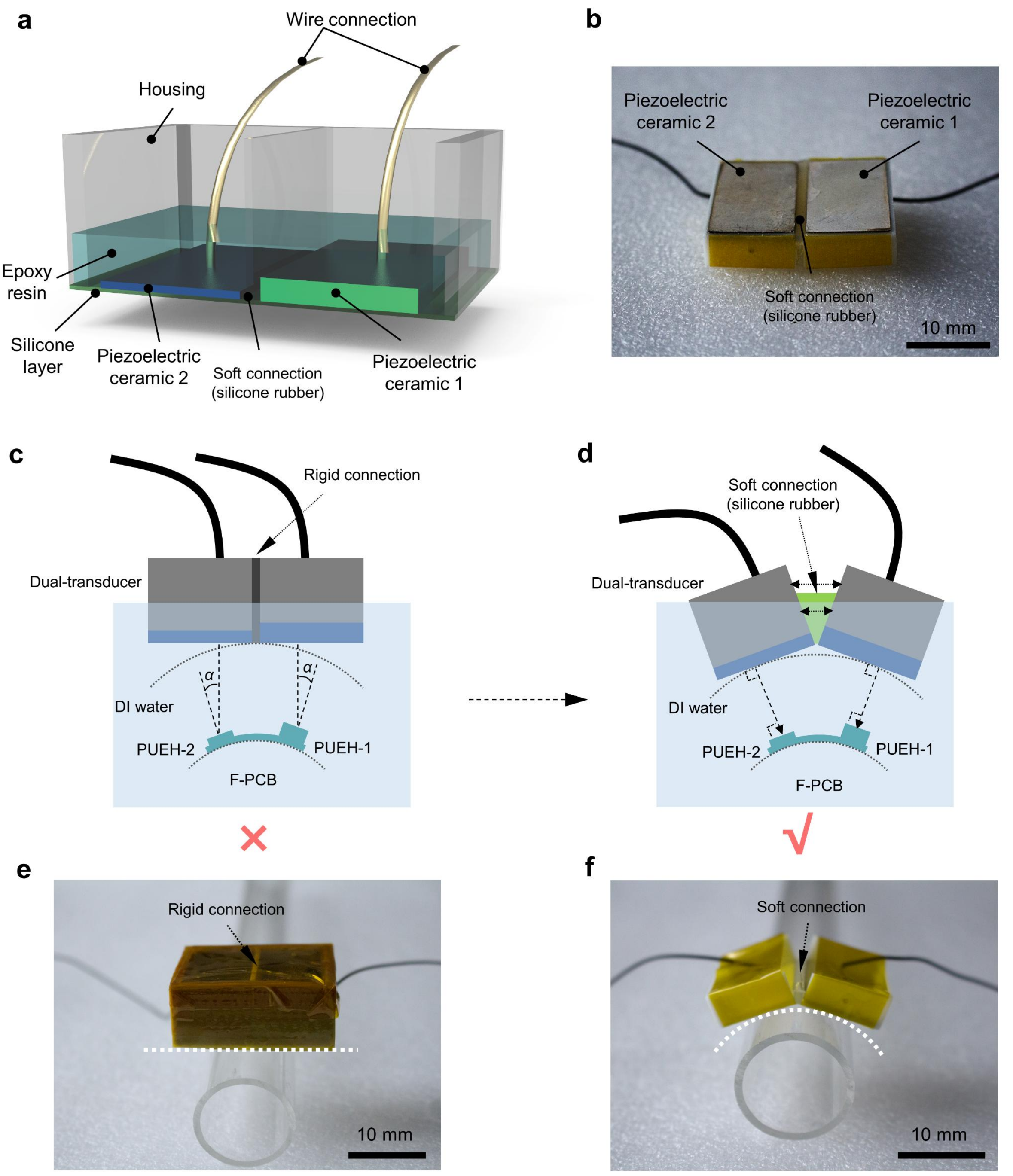

Supplementary Fig. 1 | Design and advantages of the external dual-transducer with soft connection. (**a**) The schematic layout of the dual-transducer. (**b**) Optical image of the dual-transducer. (**c,d**) Schematic showing the advantages of a soft connection (c) for the dual-transducer, compared to a rigid connection (d). (**e,f**)

Pictures showing dual-transducers with a rigid connection (e) and a soft connection (f) on a curved surface.

Two piezoelectric ceramics of different resonance frequencies (1 MHz and 3 MHz) were deployed and encapsulated to fabricate the dual-transducer to transmit the ultrasound waves. The transducer is portable and require no physical connection with the implant. In addition, our stimulator is manufactured from a flexible printed circuit board with two receiving piezo-elements at each end that is designed to accommodate the curvature required for implantation above skull. Therefore, the angle of the two elements may not be in a plane when implanted. In general, acoustic receivers usually require special angles related to the ultrasonic beam to achieve optimal performance. The maximum ultrasound intensity will be delivered to the harvester if the acoustic beam is perpendicular to the surface of piezo-elements. Otherwise, the incident ultrasonic power will be weakened if the piezo-elements are tilted at an angle to the acoustic beam. To enhance transfer efficiency and stability and provide design advantages, we designed and prepared the external dual-transducer through the use of a soft connection (silicone rubber). As a result, this dual-transducer with soft connection adapts to the curved shape and thus maintains a good angle with the respective receivers (**Supplementary Figure 1d and f**), allowing both receivers to have a more balanced output.

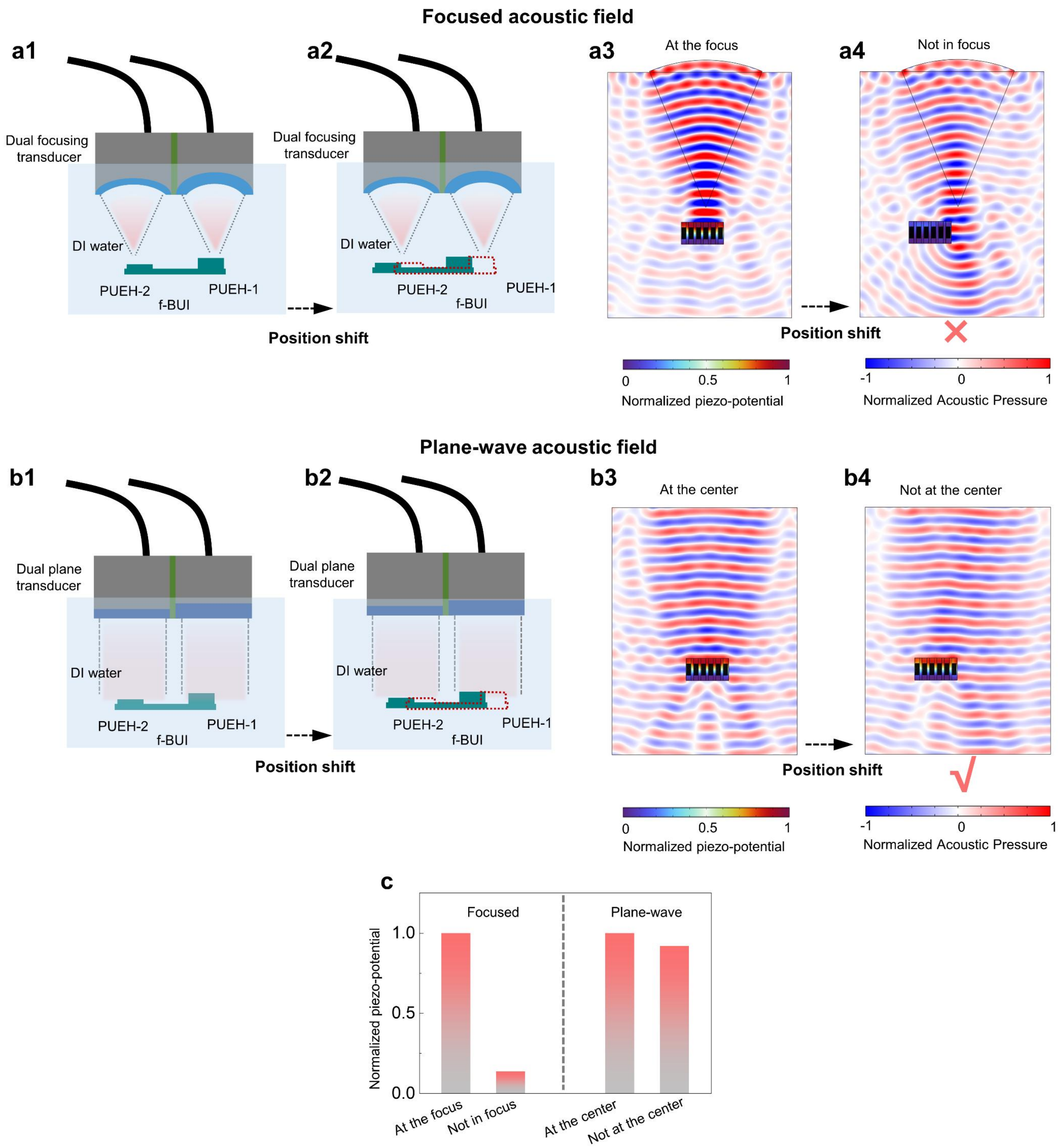

Supplementary Fig. 2 | Comparison of piezoelectric output in the focused and plane-wave acoustic fields when the harvester position is varied. (**a1,a2**) Schematic showing the change in position of the harvesters in a dual focused acoustic field. (**a3,a4**) Simulated the piezoelectric potential of a harvester as its position changes in a focused acoustic field. (**a3**) At the focus. (**a4**) Not in the focus. (**b1,b2**) Schematic showing the change in position of the harvesters in a dual plane-wave acoustic field. (**b3,b4**) Simulated piezoelectric potential of a harvester as its position changes in a plane-wave acoustic field. (**b3**) At the center. (**b4**) Not in the center. (**c**)

Comparison of the piezoelectric output as the harvester position is varied in a focused acoustic field and a plane-wave acoustic field.

For implantable applications, the acoustic field optimization needs to place primary emphasis on the challenges of transmitter-receiver alignment, especially for dual-frequency operation in this work. In general, the acoustic energy generated by the focusing transducer is focused into a small area through the confined wave beam, which can improve the magnitude of the acoustic excitation and also reduce energy dissipation, thereby increasing power conversion efficiency[1]. However, in that focused acoustic field, the focusing point is usually small (only millimeters or sub-millimeters for MHz ultrasound), and the energy rapidly decays once it moves away from the focus. The piezoelectric output of the harvester decreases substantially when it is slightly out of focus (**Supplementary Figure 2a,c**), which poses a challenge for the alignment of implanted devices, especially the dual-frequency system in this work. A slight misalignment will result in a huge difference in efficiency from channel to channel. In addition, focused ultrasound has been shown to have a therapeutic effect[2,3] and tends to confuse the electrical stimulation response by causing additional interference with our electrical stimulation devices. However, in the case of the planar transmitting transducer, the acoustic energy generated is distributed more homogeneously at its front end. Even if the position of the harvester is slightly off (off-center), its acoustically induced piezoelectric potential is not significantly attenuated (**Supplementary Figure 2b,c**). Thus, the plane configuration has a large positional tolerance, which facilitates the transmitter-receiver alignment of the dual channels in this work.

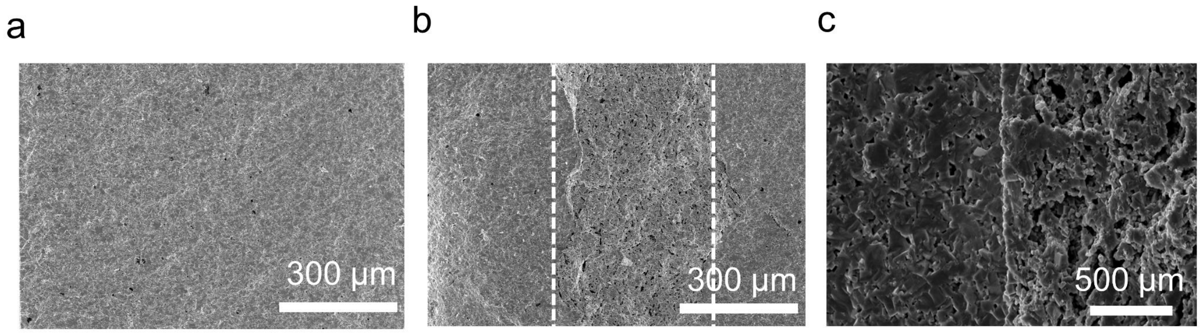

Supplementary Fig. 3 | Surface SEM images of ceramics. Surface morphology of (**a**) dense ceramic, (**b**) sandwich ceramic and (**c**) dense ceramics vs. porous ceramics, respectively. Experiments in a-c were repeated three times with similar results.

The microstructure of the sandwich ceramics clearly shows a dense-porous-dense structure, and the introduction of PS has indeed made the ceramics more porous.

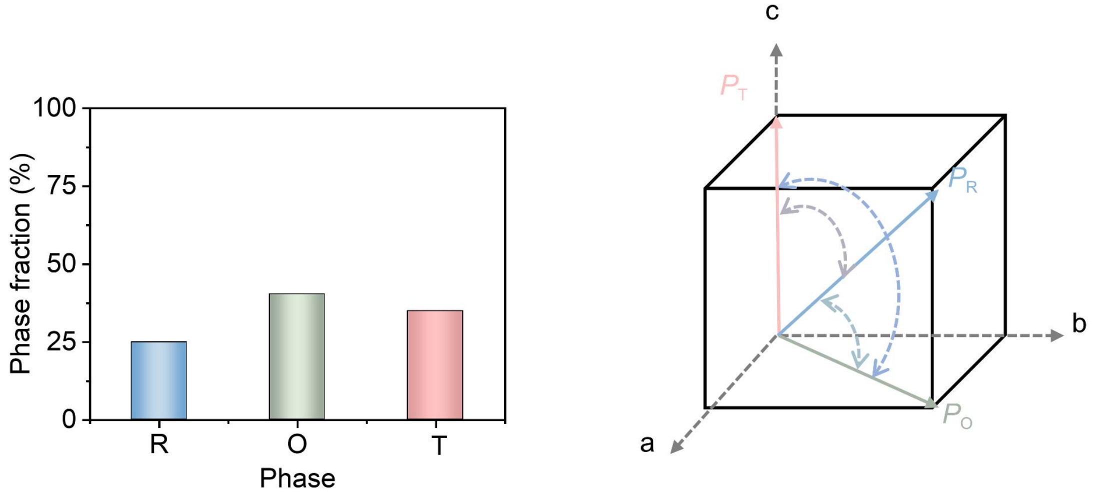

Supplementary Fig. 4 | Phase fraction of each phase of KNNS95 ceramics.

The KNNS95 ceramic exhibits R-O-T phase coexistence at room temperature with a phase fraction of 24.8%, 40.3%, and 34.9% for R, O and T phases, respectively, laying the groundwork for high piezoelectricity.

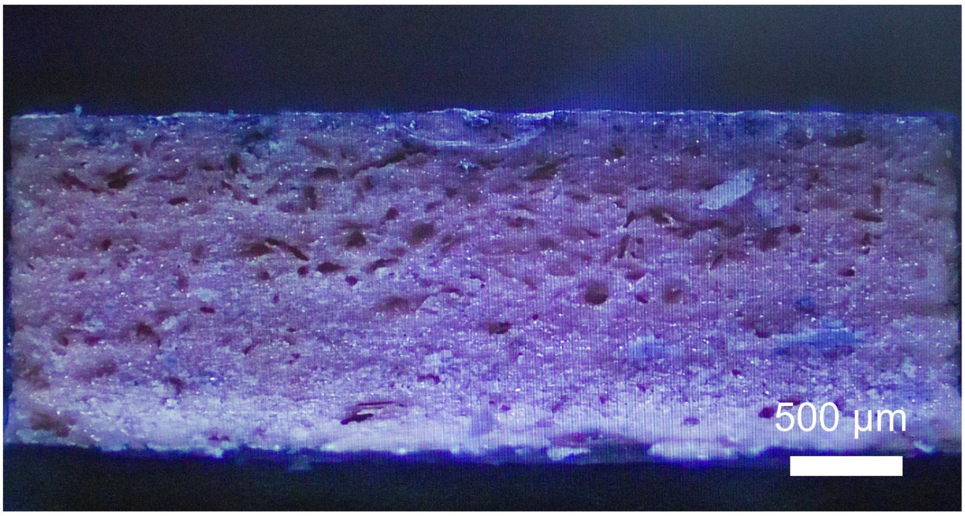

Supplementary Fig. 5 | Optical image of the porous ceramic cross-section.

The introduction of polystyrene (PS) microspheres makes the porous structure in ceramics, and we observed the infiltration of silver electrodes in the porous ceramic cross-section, which is detrimental to subsequent device preparation. Experiments were repeated three times with similar results.

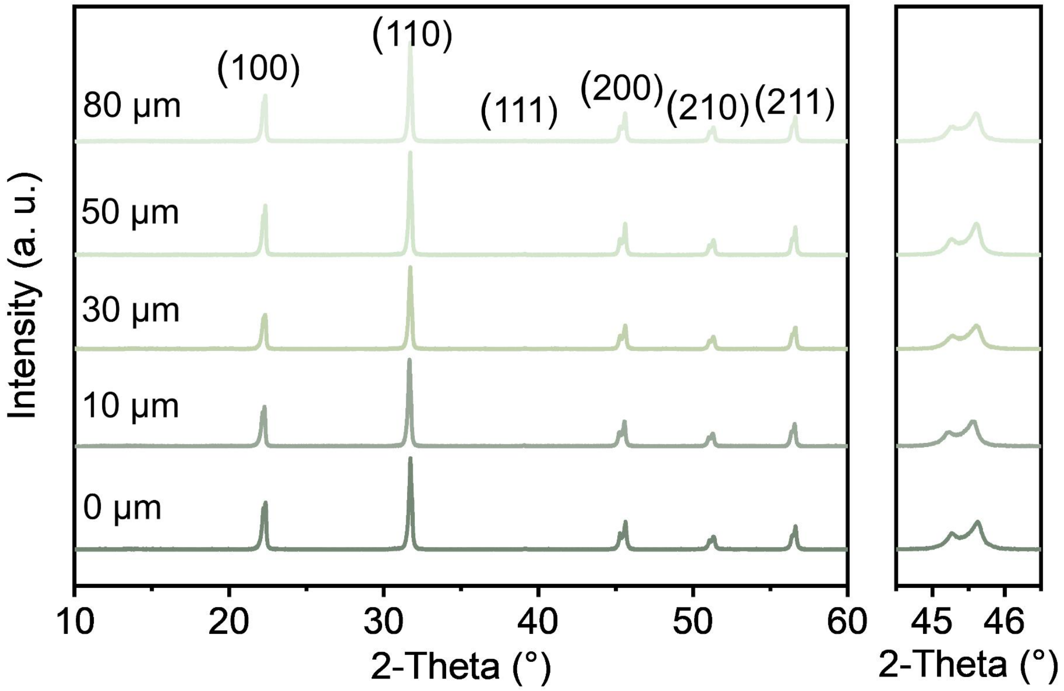

Supplementary Fig. 6 | Room temperature XRD patterns and magnified (200)pc diffraction peaks of sandwich ceramics with varying diameters.

The introduction of PS microspheres did not affect the microstructure of the ceramics.

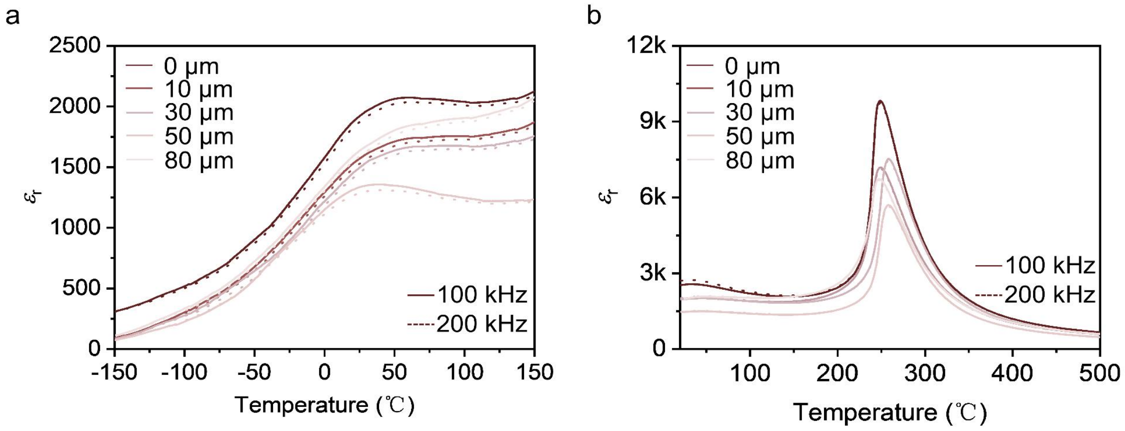

Supplementary Fig. 7 | Temperature-dependent dielectric constants of sandwich ceramics with varying diameters. Measuring from (**a**) -150 ℃ to 150 ℃ and (**b**) 30 ℃ to 500 ℃ at different frequencies.

The introduction of PS did not significantly change the phase transition temperature of the ceramics, but it did reduce the dielectric value. Comparing the different sizes, the 50 um one has the lowest dielectric constants, which is consistent with our experimental results.

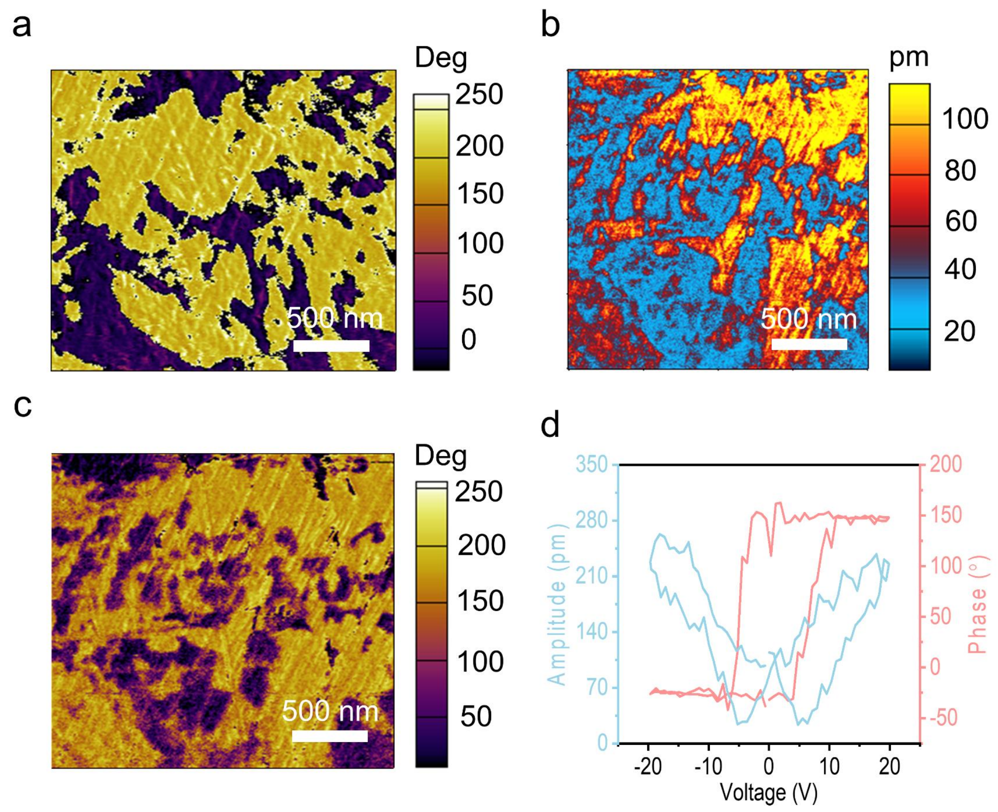

Supplementary Fig. 8 | Domain structural characterization of the ceramics measured by PFM. (**a**) phase image of dense ceramics. (**b**) amplitude, (**c**) phase and (**d**) SS-PFM curves of porous ceramics, respectively.

The introduction of PS reduces the piezoelectric response of the ceramic, but still exhibiting typical butterfly amplitude curves and nearly rectangular phase loops, indicating that the design of sandwich structure is reasonable, again.

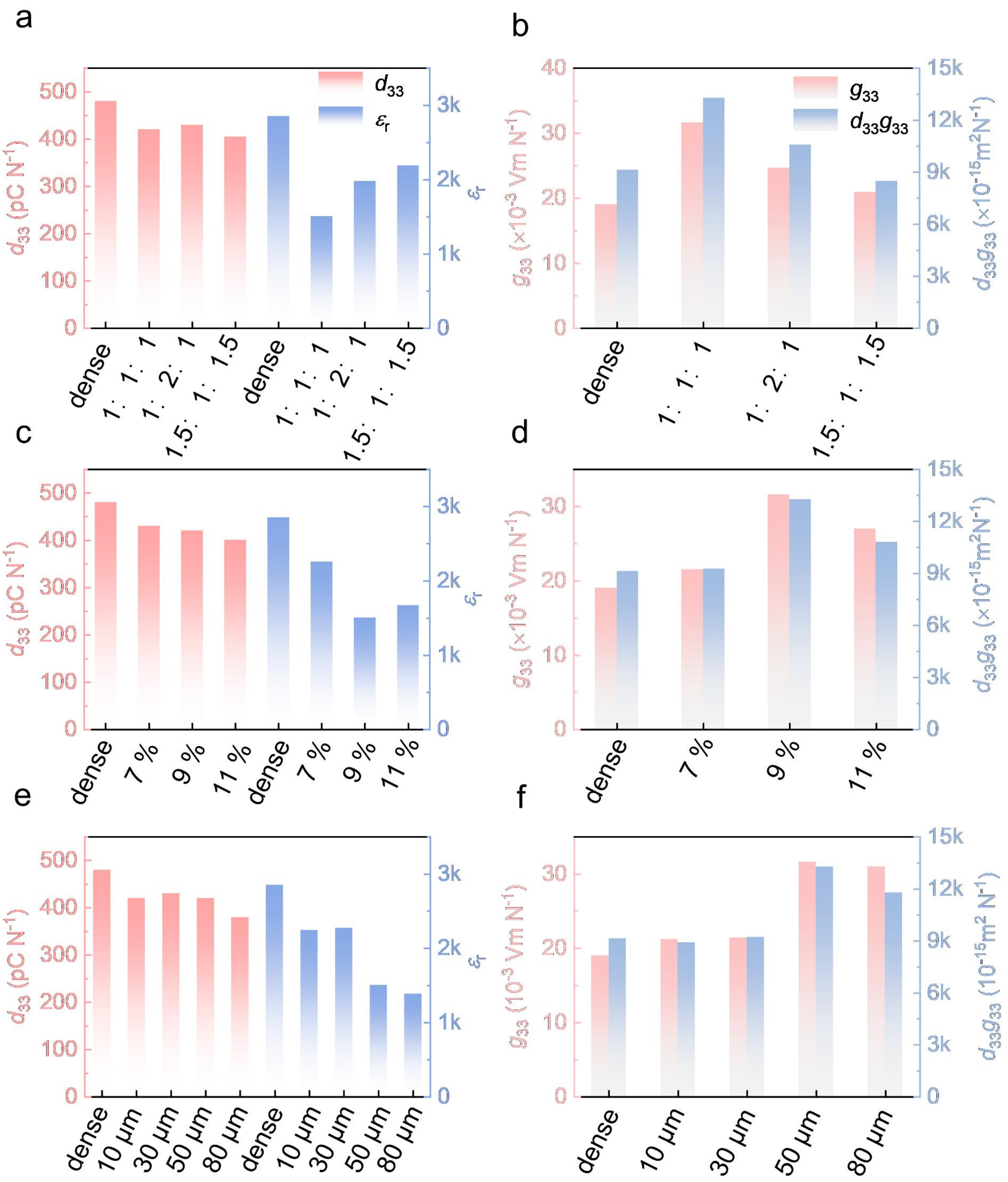

Supplementary Fig. 9 | Electrical properties of ceramics with different conditions. Properties of d_{33} and ε_r with (**a**) different mass ratio for dense and porous layers, (**c**) different proportion for PS microspheres, (**e**) different diameters for PS microspheres. Properties of g_{33} and $d_{33} \times g_{33}$ with (**b**) different mass ratio for dense and porous layers, (**d**) different proportion for PS microspheres, (**f**) different diameters for PS microspheres.

For optimal performance of sandwich construction ceramics, electrical properties of different layer thickness ratios, different diameters and scales of PS were sorted out to obtain optimal components. The optimum $d_{33} \times g_{33}$ value of 13272 × 10^{-15} m^2 N^{-1} when the layer thickness ratio is 1:1:1, the diameter is 50 μm and the scale is 9 wt.%, and following-up experiments on this basis.

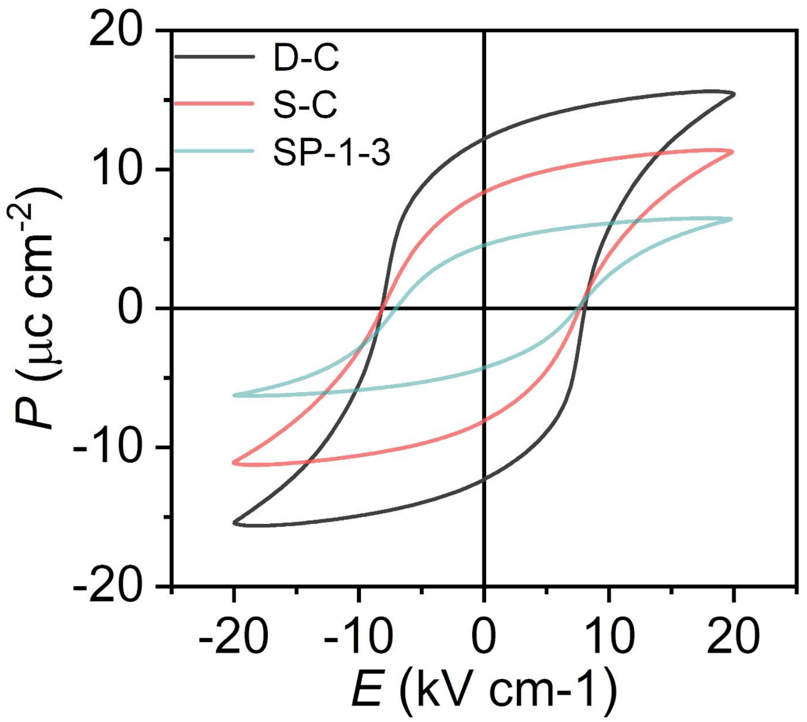

Supplementary Fig. 10 | Polarization-electric field (*P-E*) hysteresis loops of dense KNNS95 ceramics (D-C), sandwich ceramics (S-C), and piezoelectric sandwich porous 1-3 (SP-1-3) composites.

Because of the introduction of air and epoxy polymers, which are both without ferroelectric polarization, the ferroelectricity of the sandwich ceramics and SP-1-3 composites is reduced compared to the dense KNNS95 ceramics.

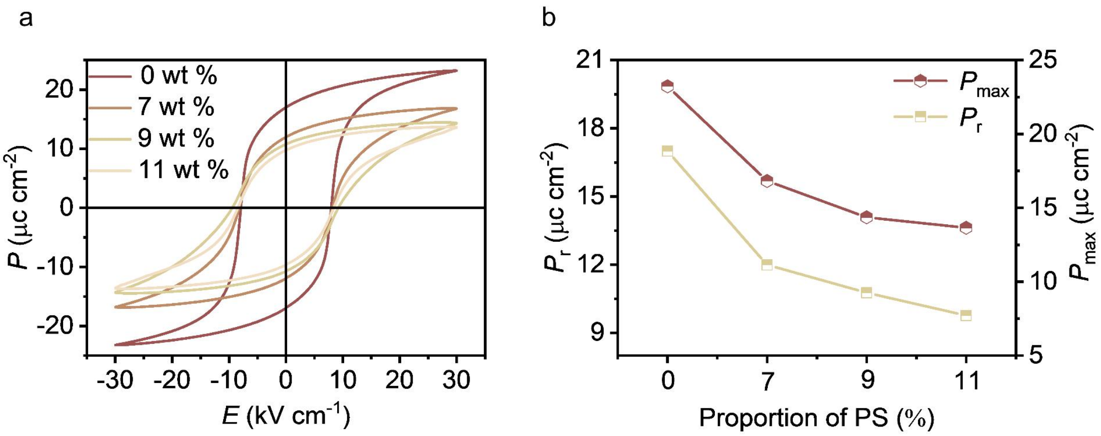

Supplementary Fig. 11 | Ferroelectric properties of the sandwich ceramics with varied proportion of PS. (**a** and **b**) *P*-*E* loops (a) and P_r and P_{max} (b) varying with proportion of PS.

Accompanied by the introduction of different proportions of PS, values of P_r and P_{max} of the ceramics were reduced compared to the original dense ceramics, but an inflection point was observed from 9%.

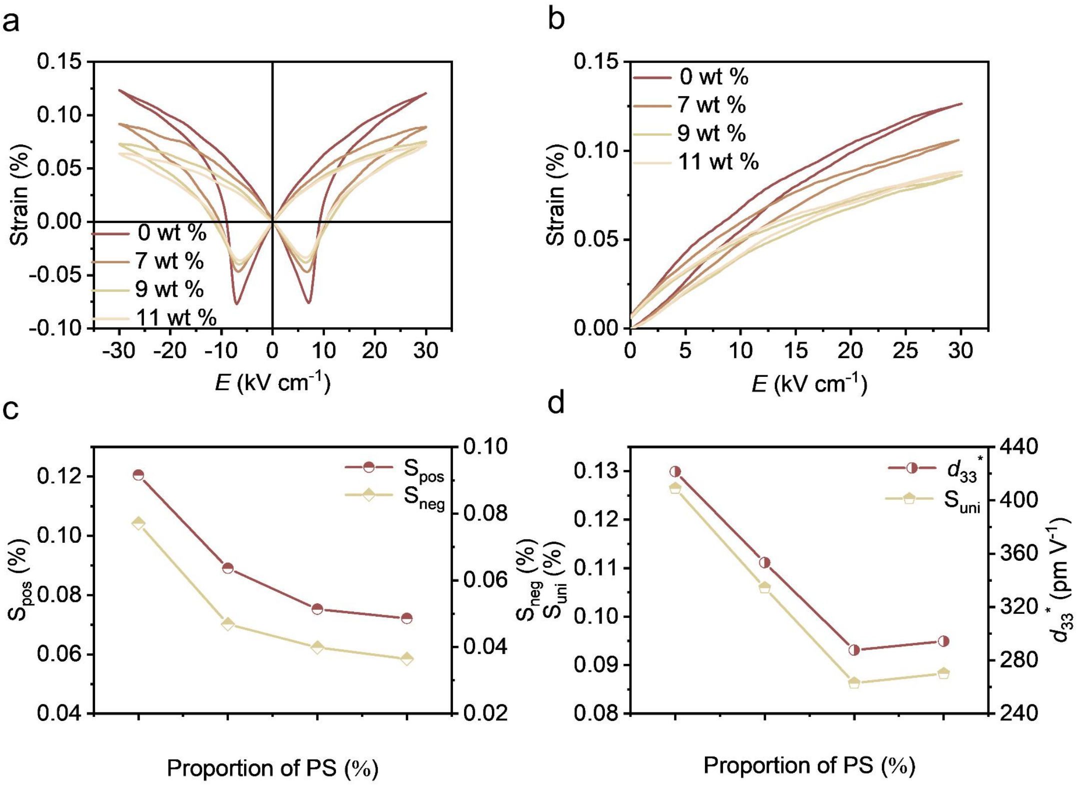

Supplementary Fig. 12 | Strain properties of the sandwich ceramics with varied proportion of PS. (**a**) Bipolar strain curves, (**b**) Unipolar strain curves, (**c**) S_{pos} and S_{neg} and (**d**) d_{33}* and S_{max} varying with proportion of PS.

Together with other electrical properties, we determined the optimal PS doping ratio.

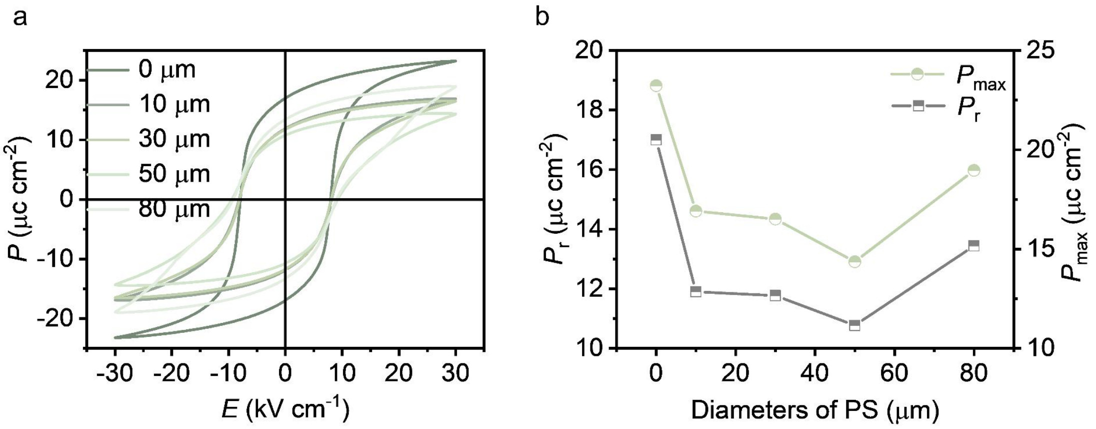

Supplementary Fig. 13 | Ferroelectric properties of the sandwich ceramics with varied diameters of PS. (**a**) *P-E* loops and (**b**) P_r and P_{max} varying with diameters of PS.

Accompanied by the introduction of different proportions of PS, the strain properties of the ceramics were reduced compared to the original dense ceramics, but an inflection point was observed from 9%.

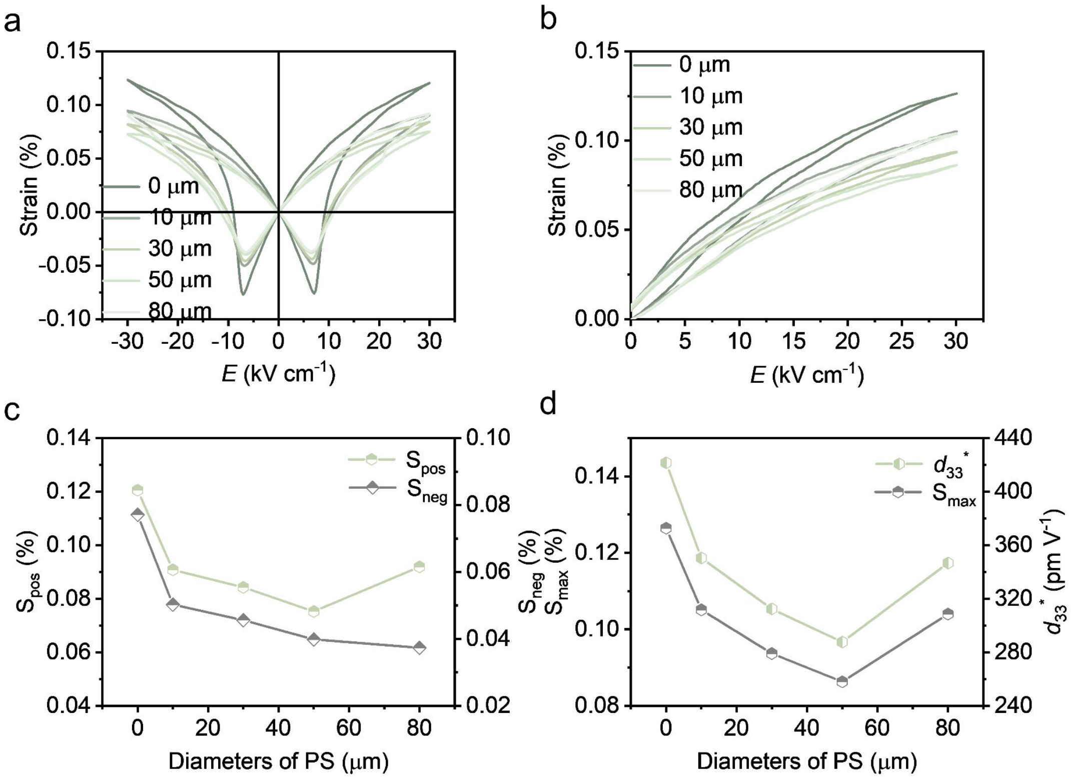

Supplementary Fig. 14 | Strain properties of the sandwich ceramics with varied diameters of PS. (**a**) Bipolar strain curves, (**b**) Unipolar strain curves, (**c**) S_{pos} and S_{neg} and (**d**) d_{33}* and S_{max} varying with diameters of PS.

Together with other electrical properties, the optimal diameter of PS is 50 μm.

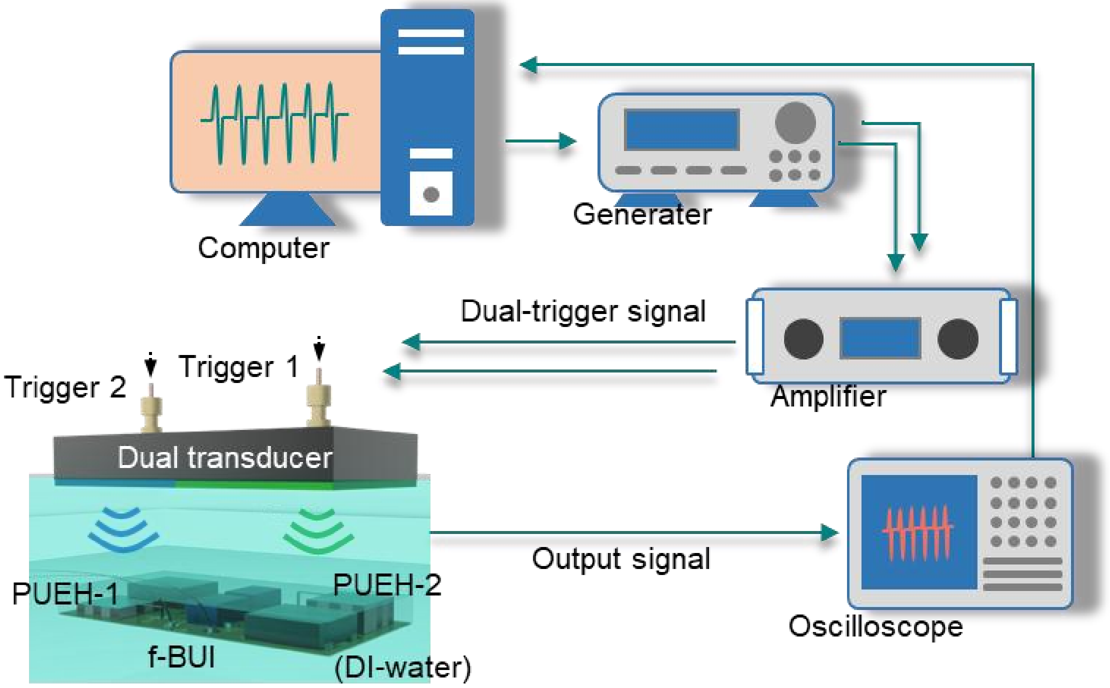

Supplementary Fig. 15 | Schematic of the measurement setup for ultrasound energy transfer system.

When we give a dual-trigger signal on the dual-transmitter, the acoustic pressures will apply to two PUEHs in the device through the propagation of the ultrasonic waves in water. Due to the acoustic pressure, a corresponding strain is generated in the piezoelectric composites, accompanied by a piezoelectric potential excited by the piezoelectric effect. It then turns into an electrical signal output that flows through the external circuit so that we can detect the corresponding electrical response.

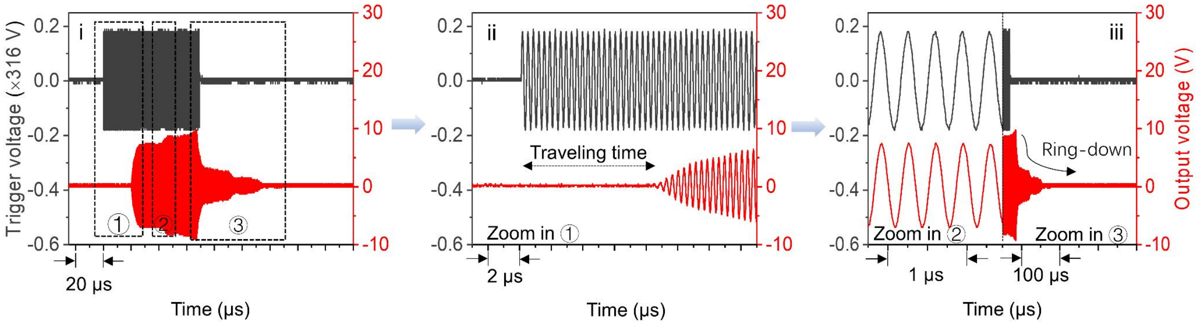

Supplementary Fig. 16 | Comparison between the trigger signals and output signal in PUEH-2. i) whole, ii) and iii) zoom in locally for the ~3 MHz one from the f-BUI.

Both of the piezoelectric ultrasound energy harvesters (PUEH-1 and PUEH-2) with different resonance frequencies (~1 MHz one and the ~3 MHz one) are validated that the output signals are indeed excited by the ultrasound generated from the transmitter, instead of other interfering signals.

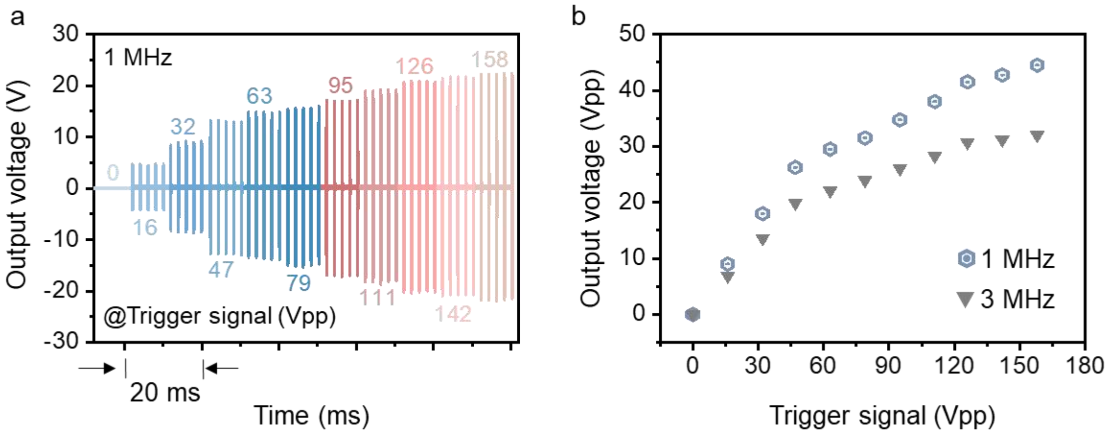

Supplementary Fig. 17 |. Performance characterization of the PUEH-1. (**a**) Output voltage amplitude with varied trigger voltages at 1 MHz-transmission. (**b**) Output voltage values with varied trigger voltages.

Taking the 1 MHz-transmission as an example, the output voltages increase first and then decrease ranging from 0.5 to 1.7 MHz, getting its maximum value at 1.06 MHz. The amplitudes increased with increasing input voltage, with a voltage efficiency of ~53% as the input amplitude is below 47 Vpp. However, when the input amplitude exceeds 47 Vpp, the rise rate drops and there is no longer such a significant rise, indicating a slow-growth tendency of the output voltage, but reaching ~44.5 Vpp as the trigger voltage is 158 Vpp. That is due to the thermal effect of the equipment and the cavitations of water caused by the ultrasound resulting in additional energy losses and reduced efficiency. For the 3 MHz-transmission channel, optimal transmission frequency at ~3 MHz. The output voltage trend is similar to 1-MHz channel, but the voltage efficiency is slightly lower than 1-MHz channel due to higher attenuation.

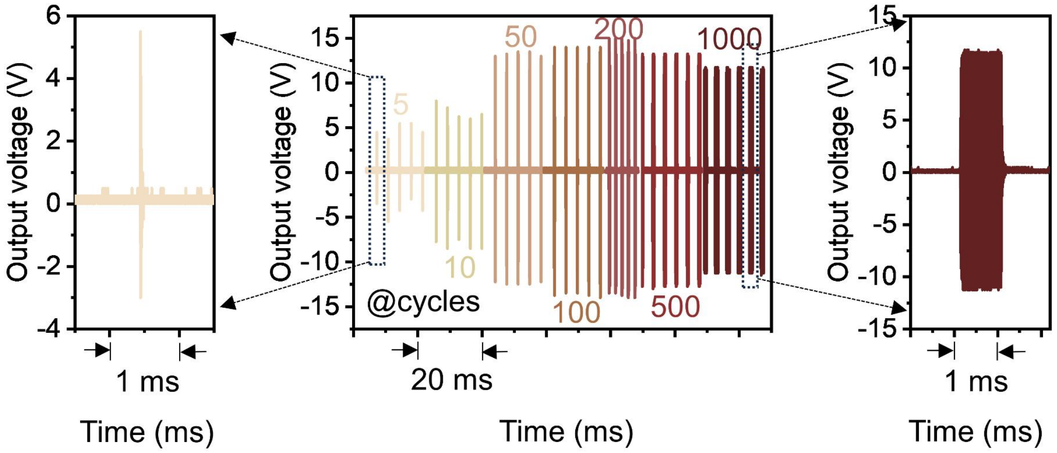

Supplementary Fig. 18 | Output voltages under the excitation of trigger signals with different cycles.

The voltage outputs increase first and then decrease ranging from 5 to 1000 cycles. These results demonstrate that the ultrasound-induced energy can be flexibly adjusted by changing the duty cycle.

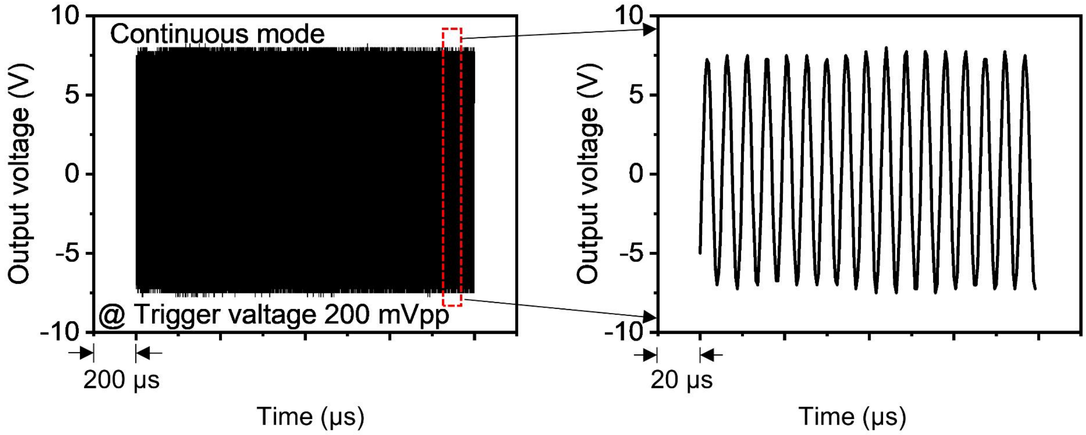

Supplementary Fig. 19 | Output voltages of the sample measured in a continuous mode.

A continuous sinusoidal signal is switched to trigger the transmitter and the output generated by the sample is correspondingly a continuous sinusoidal. The output voltage of the sample shows no degradation during the continuous mode measurement, suggesting the reliability of ultrasonic energy transmission.

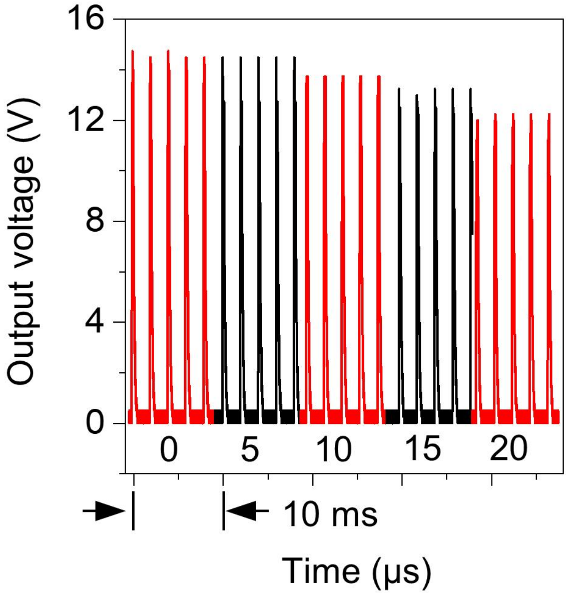

Supplementary Fig. 20 | Characterization of the ultrasound-induced electrical outputs of f-BUI at 1 MHz channel in the ex vivo porcine experiment. The output voltages of f-BUI testing under varying thickness (0 mm, 5 mm, 10 mm, 15 mm, and 20 mm) of porcine.

Owing to the attenuation and reflection of the ultrasound, the output voltage decreases with the increasing thickness (take the ~1 MHz one).

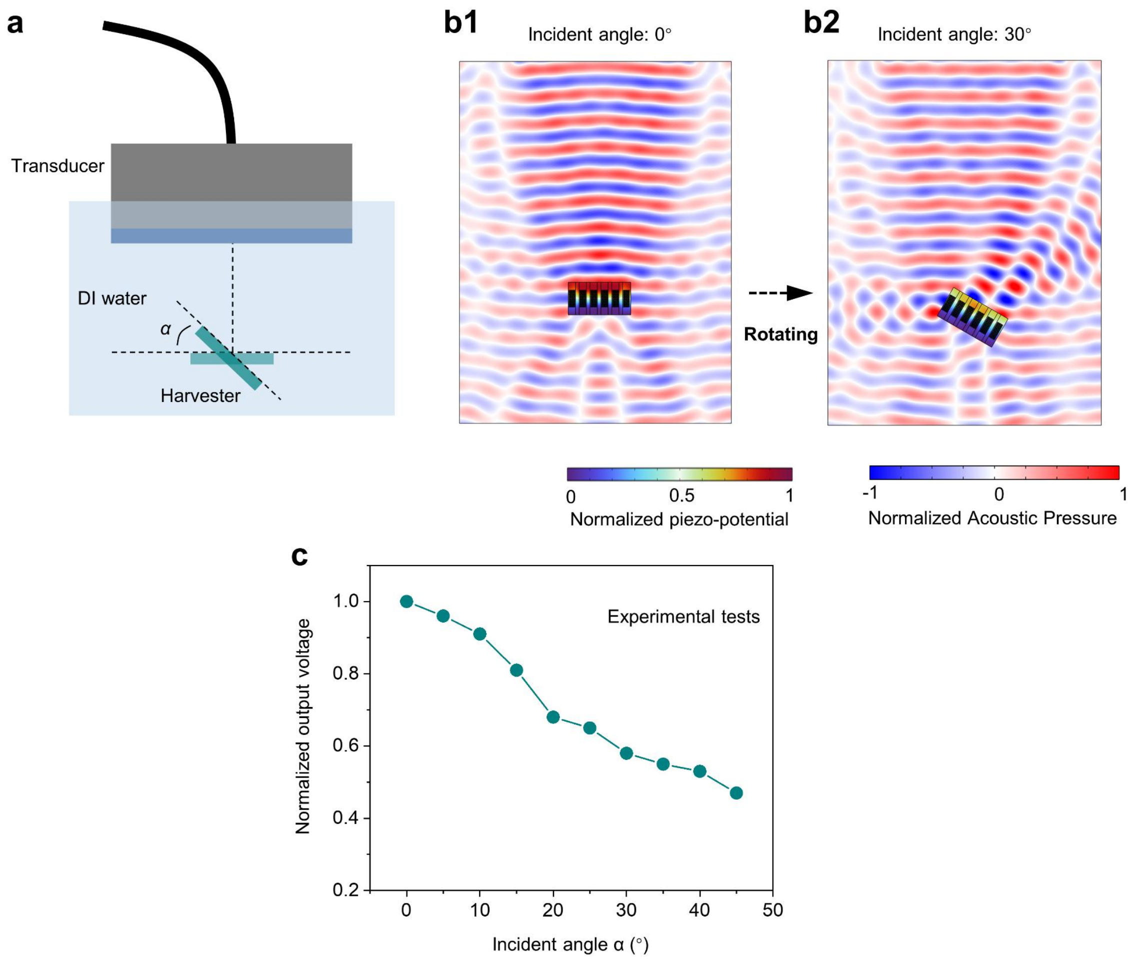

Supplementary Fig. 21 | Relationship between the output of the harvester and the incidence angle of the ultrasound wave. (**a**) Schematic showing the experimental test. (**b**) Simulated piezo-potential of a SP-1-3 piezo-harvester under ultrasonic excitation when the incident angle is 0° (b1) and 0° (b2). (**c**) Variation of the output voltage of the harvester measured at different US incident angles.

In general, acoustic receivers usually require special angles related to the ultrasonic beam to achieve optimal performance. The maximum ultrasound intensity will be delivered to the harvester if the acoustic beam is perpendicular to the surface of piezo-elements. Otherwise, the incident ultrasonic power will be weakened if the piezo-elements are tilted at an angle to the acoustic beam.

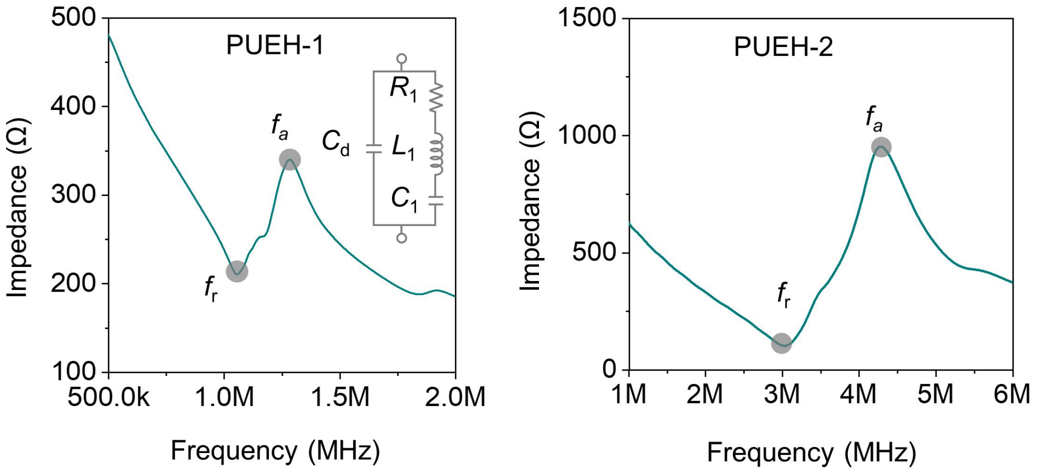

Supplementary Fig. 22 | Impedance spectra of two S-1-3 PUEHs with different resonance frequencies (1 MHz and 3 MHz). Inset: equivalent *RLC* circuit diagram of the piezo-element.

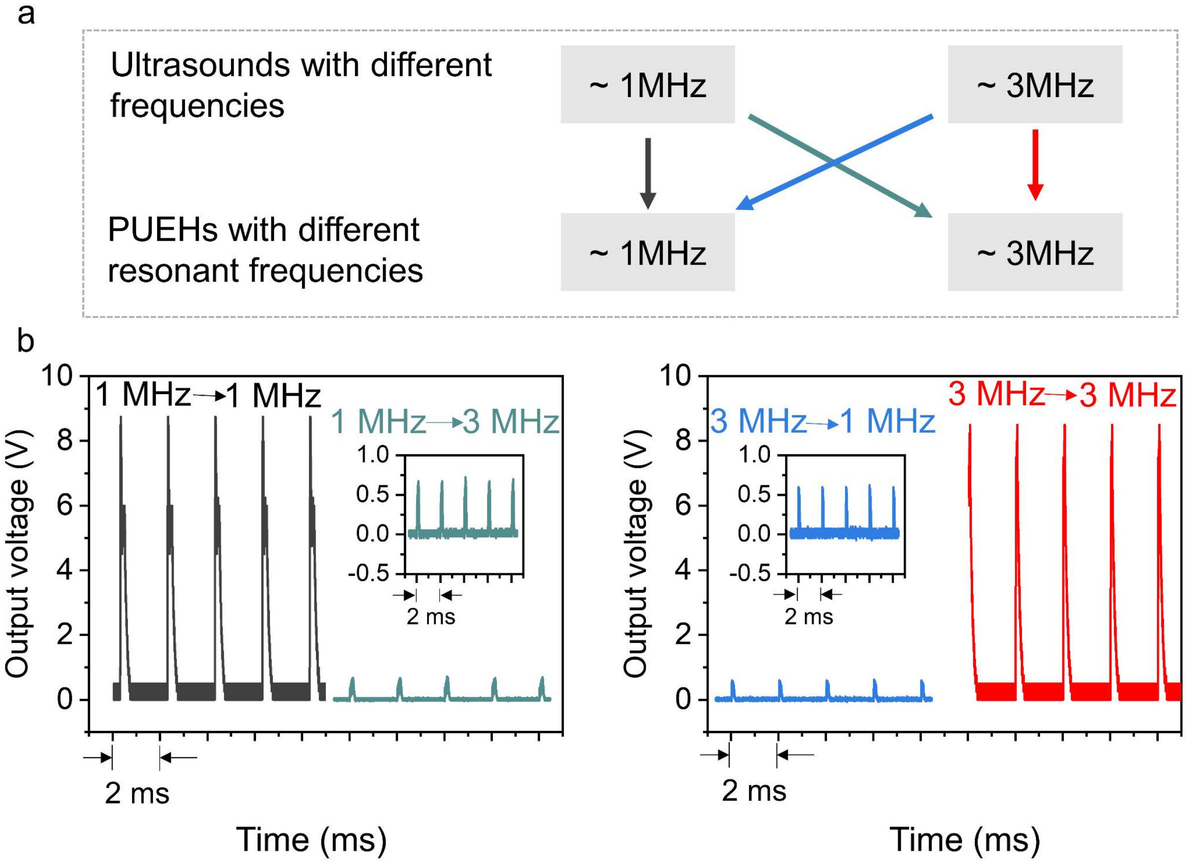

Supplementary Fig. 23 | Output performance comparison of two harvesters with different frequencies. (**a**) Schematic diagram of the ultrasounds with frequencies of 1 MHz and 3 MHz to excite the two harvesters with the resonant frequencies of 1 MHz and 3 MHz, respectively. (**b**) Rectified output voltage of two harvesters with the resonant frequencies of 1 MHz and 3 MHz excited by different frequencies.

PUEHs get excellent output performance when the inducing ultrasound near their own resonant frequency. However, when excited by ultrasound of the other’s ultrasound resonant frequency, their output voltages are almost negligible, indicating that they are not distracted by each other's stimulus signals when they are at work, and it also proves the feasibility of our idea of biphasic stimulation.

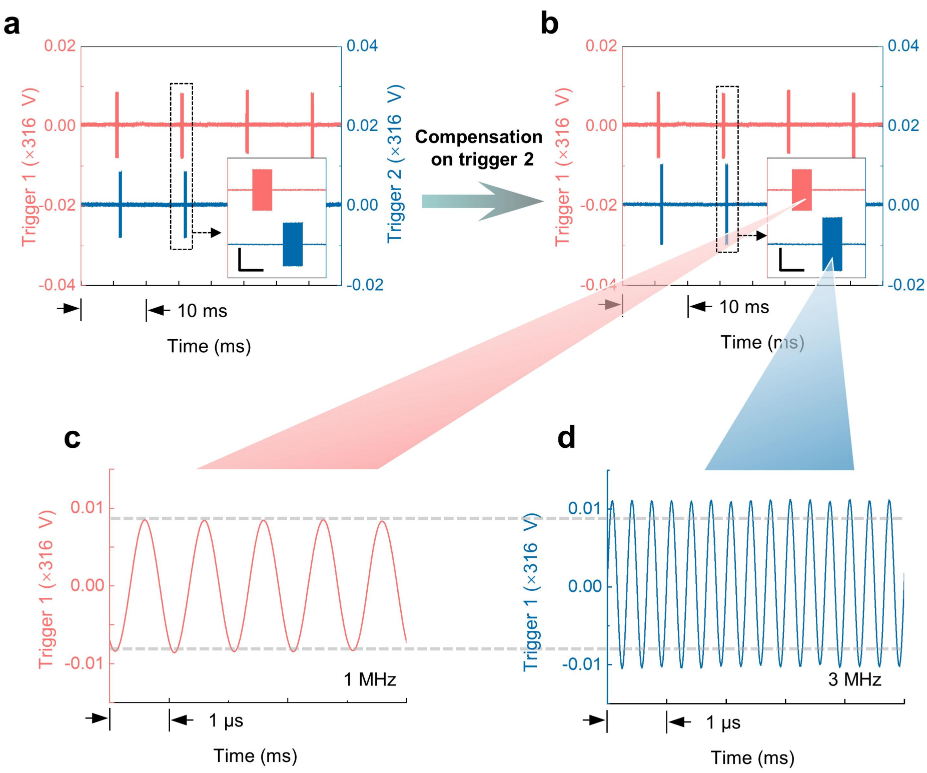

Supplementary Fig. 24 | Dual-alternating current (AC) trigger waveforms. (**a**) Trigger 1 and trigger 2 waveforms without compensation. (**b**) Trigger 1 and trigger 2 waveforms with compensation. The inset shows an enlargement of the trigger waveforms. Scale bars, 0.02 V and 500 μs. (**c,d**) Enlargement of the trigger waveforms in (b).

The trigger 1 (frequency: 1 MHz, pulse length: 400 μs, pulse period: 100 Hz) and trigger 2 (frequency: 3 MHz, pulse length: 400 μs, pulse period: 100 Hz, pulse delay: 200 μs) are turned on simultaneously at their resonance frequencies, which were generated by the function generator, then amplified by an amplifier (55 dB gain) and delivered to the dual transducer. To effectively compensate for the imbalance in charge, we increased the trigger signal (~22%) for the 3-MHz channel. From the test results (**Fig. 3k**, red line), the output pulses can be adjusted to balance, thus providing better assurance for subsequent stimulation applications.

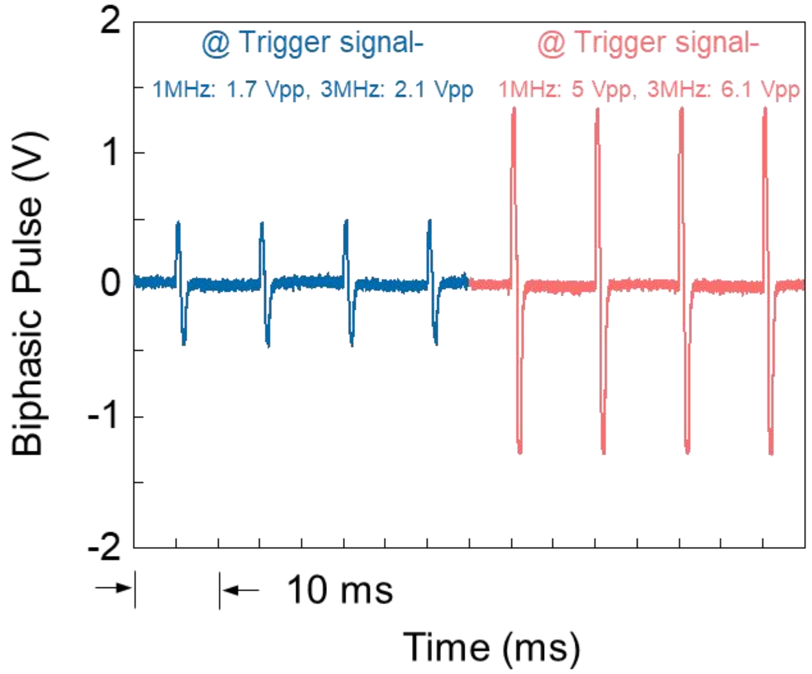

Supplementary Fig. 25 | Biphasic stimulus pulse generated by the f-BUI, where the amplitudes of biphasic stimulation waveform can be modulated by adjusting the trigger voltages of both channels simultaneously.

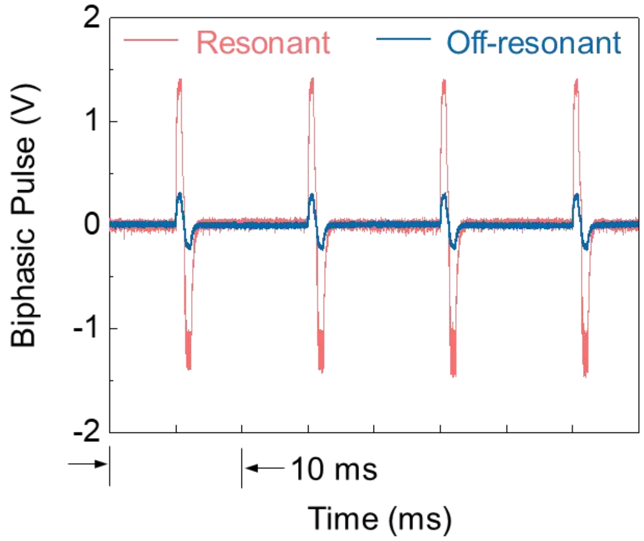

Supplementary Fig. 26 | Biphasic stimulus pulse generated by the f-BUI, where the amplitudes of biphasic stimulation waveforms is significantly different under resonant and non-resonant operation. The pulse amplitude attenuates significantly once they are switched to the off-resonance operation.

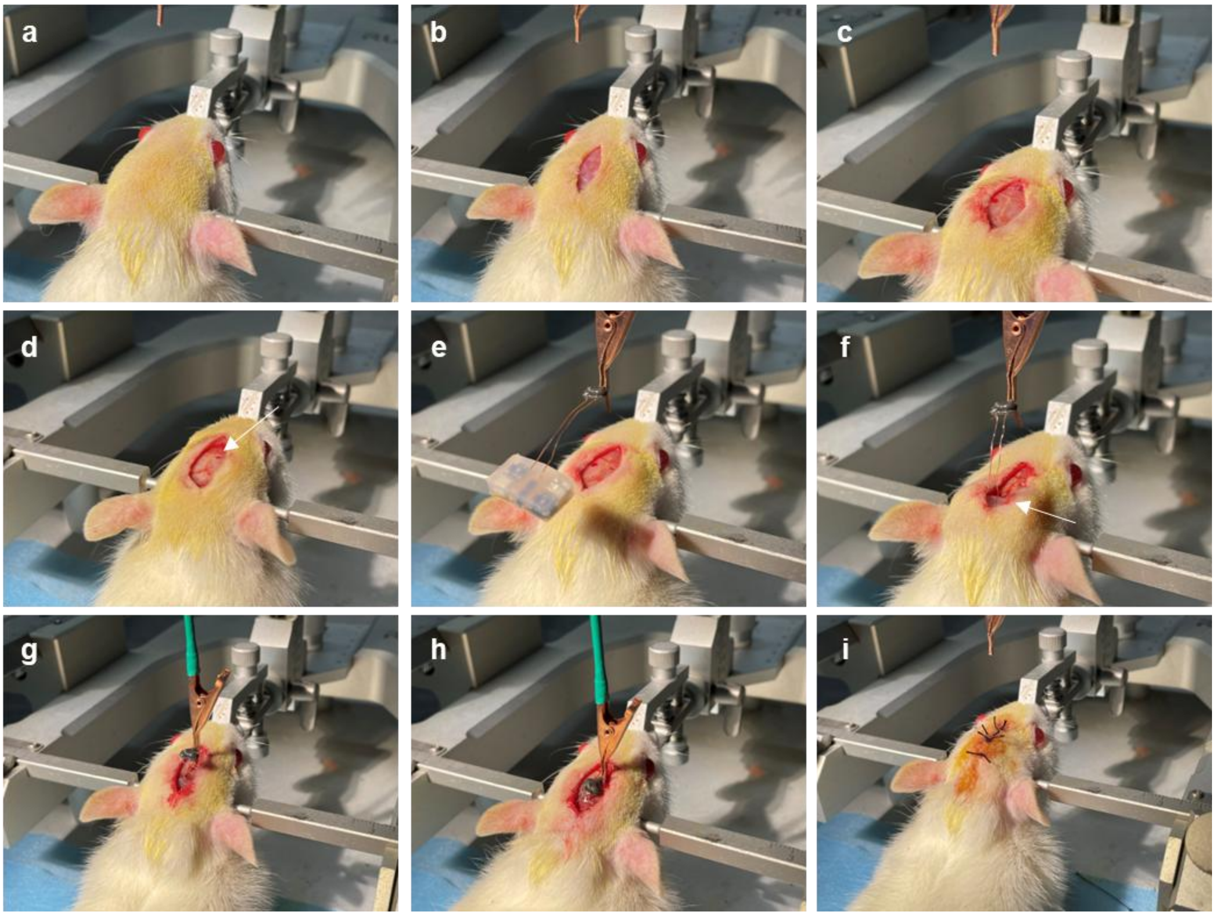

Supplementary Fig. 27 | Implantation procedure for the f-BUI for rats. (**a**) Put the rat on a stereotaxic apparatus. (**b**) Cut the skin incisions on the head. (**c**) Expose and clean the skull. (**d**) Confirm the and drill injection holes in the skull according to the implantation coordinates. (**e**) Clamp the electrode with a clip and place it above the skull. (**f**) Implant the device subdermally through head incision. (**g**) Inject the electrode. (**h**) Fix the electrode with dental cement. (**i**) Follow institutional guidelines for post-surgical procedures and monitoring after suturing the skin incision.

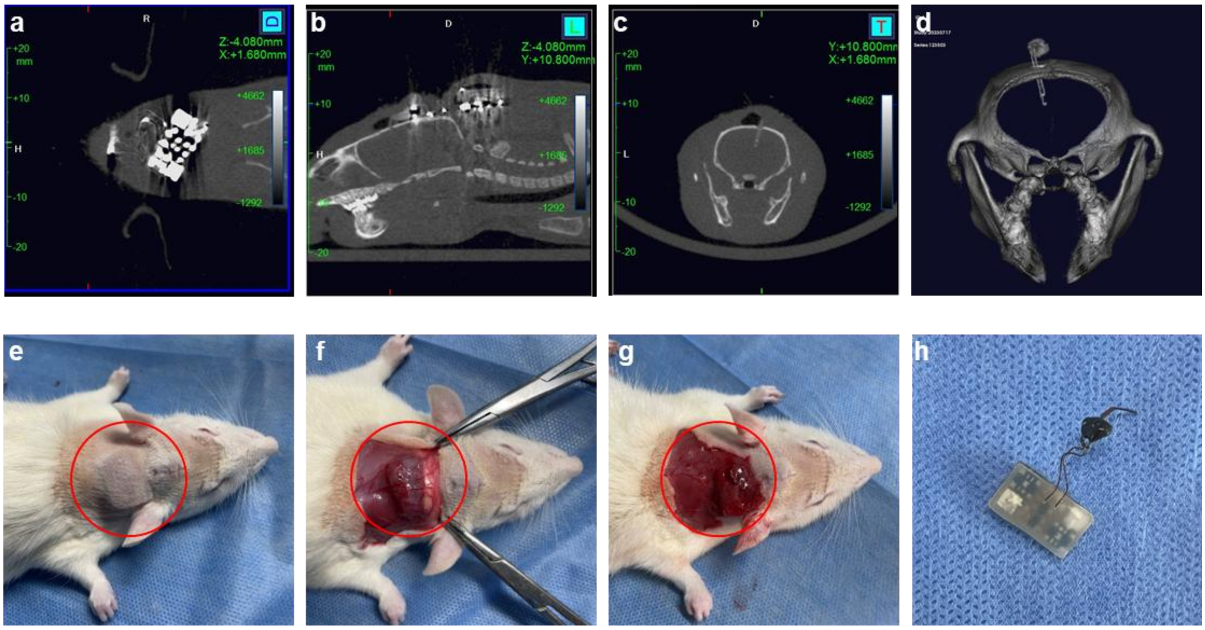

Supplementary Fig. 28 | Micro-CT imaging and photographs of f-BUI in the rat brain. (**a**) Coronal, (**b**) sagittal orientation and (**c**) axial orientation. (d) 3D rendering image of the electrode in the rat brain. (**e**) Photo of rat skin under which the f-BUI device is implanted. (**f**) and (**g**) Photos of the rat muscle tissue on which the f-BUI device is implanted. (**h**) Photo of the implanted f-BUI device after its extraction from the rat after 30 days.

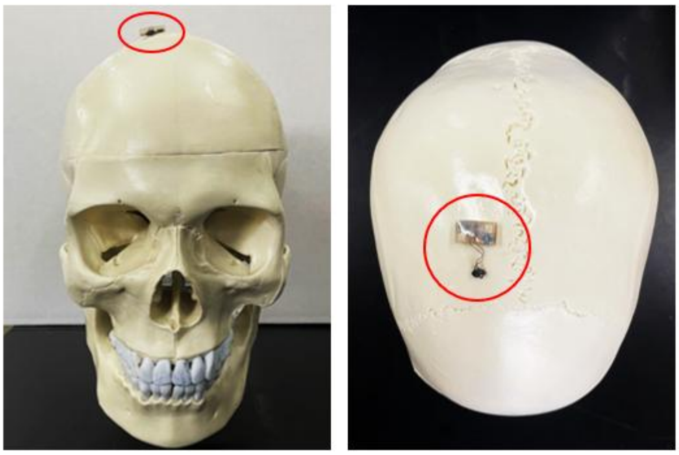

Supplementary Fig. 29 | Front view and top view of skull phantom with the top f-BUI device.

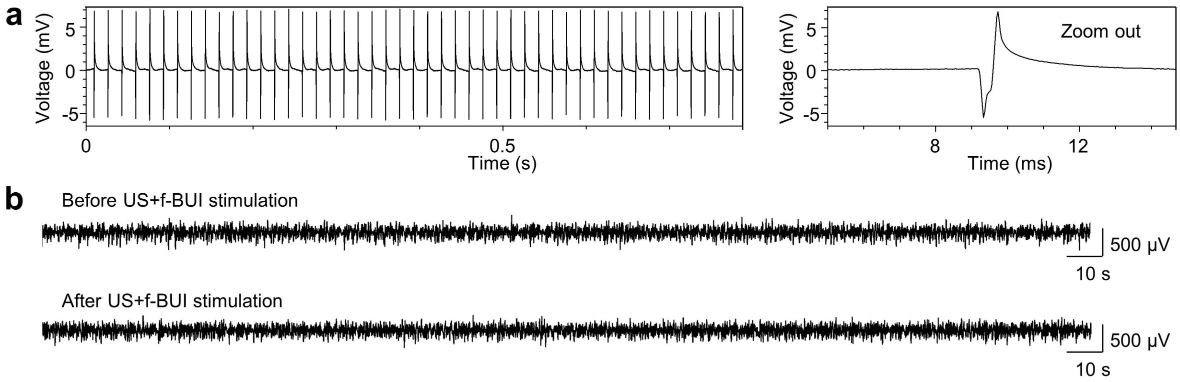

Supplementary Fig. 30 | ECoG signals record in Sham group treated by US+f-BUI. (a) Representative ECoG signals record during the US+f-BUI stimulation in Sham group. (b) ECoG signals record in the Sham+US+f-BUI group before and after US+f-BUI stimulation.

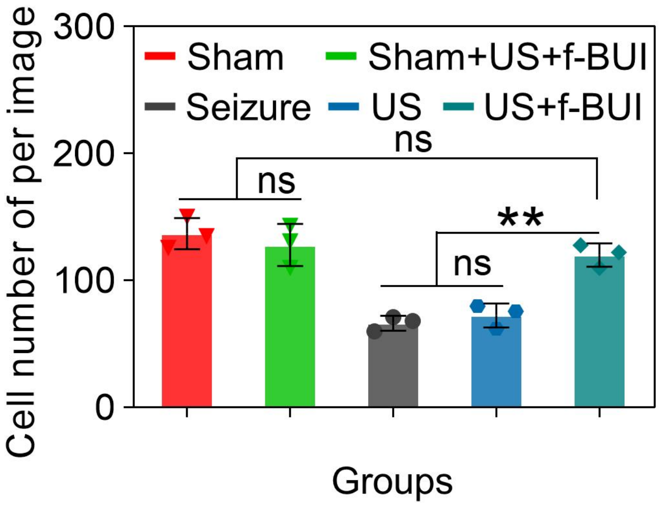

Supplementary Fig. 31 | Quantification of the cells number in the CA3 region after different treatment (*n* = 3 biologically independent samples). Statistical significance was determined by One-ANOVA with Tukey's multiple comparisons (***p*<0.01 and ns, not significant); data were presented as mean values ± standard deviations (SD); error bars = SD.

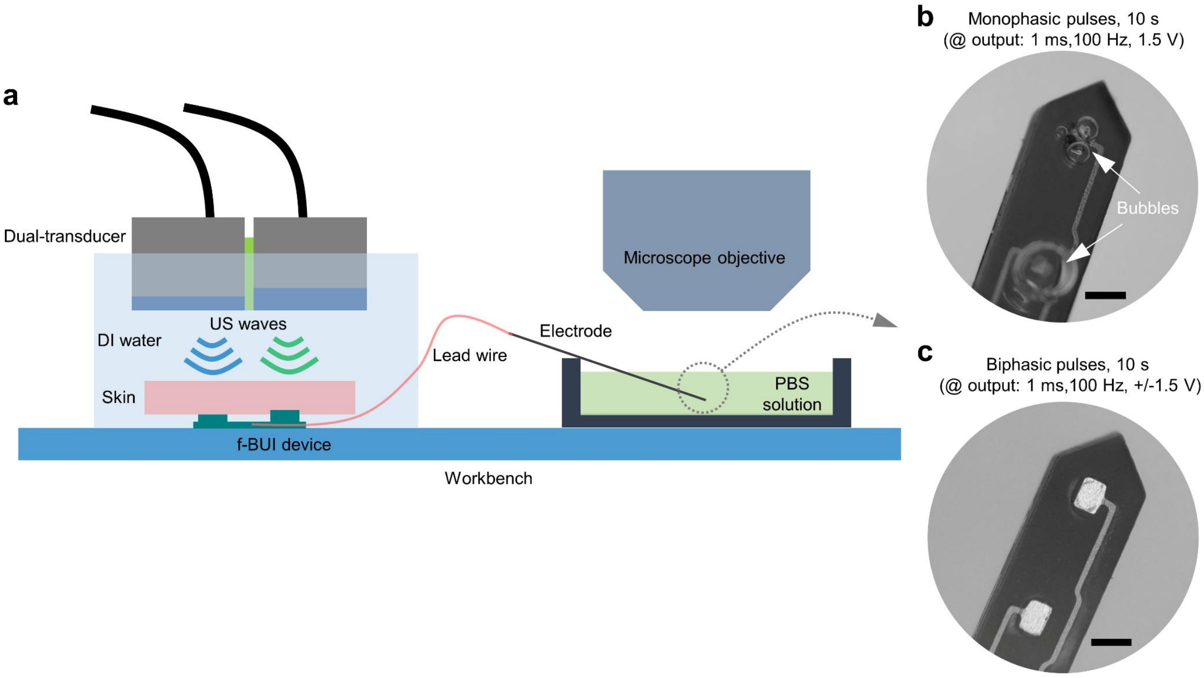

Supplementary Fig. 32 | Comparison of electrolysis experiments by using monophasic pulses and biphasic pulses. (**a**) Schematic diagram of electrolysis experiment of f-BUI device. (**b**) Electrode under the microscope during biphasic operation (10 s). (c) Electrode under the microscope during monophasic operation (10 s). Electrolysis occurs on an electrode in PBS solution, as evidenced by gas bubbles. Scale bars are 200 μm. Experiments in b,c were repeated three times with similar results.

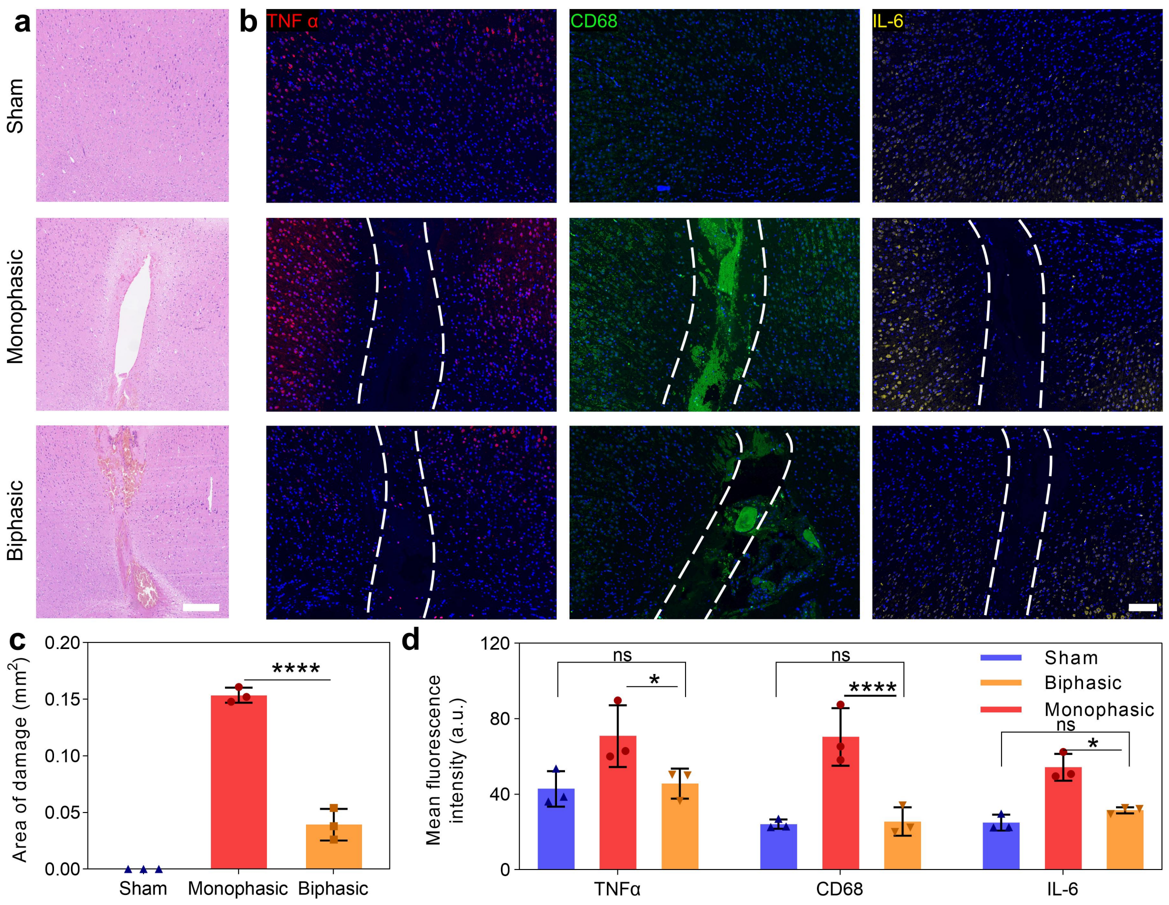

Supplementary Fig. 33 | Comparison of stimulation experiments in vivo by using monophasic pulses and biphasic pulses. (**a**) Representative H&E staining of rat brain by monophasic and biphasic stimulation (*n* = 3 biologically independent samples). Scale bar is 250 μm. (**b**) Representative immunofluorescence staining of inflammatory markers (TNFα, CD68, and IL-6) of stimulation site (*n* = 3 biologically independent samples). Scale bar is 100 μm. Experiments in (**a**,**b**) were repeated three times with similar results. (**c**) Quantitative analysis of area of damage resulting from different external electrical stimulation (*n* = 3). (**d**) Quantitative mean fluorescence intensity of TNFα, CD68, and IL-6 in the stimulation site (*n* = 3). (**c,d)** Biologically independent samples; statistical significance was determined by One-ANOVA with Tukey's multiple comparisons (****p<0.0001, ***p<0.001, **p<0.01, *p<0.05, and ns, not significant); data were presented as mean values ± standard deviations (SD); error bars = SD.

Supplementary Tables

Supplementary Tab. 1 | Comparison of device dimensions for brain implantation

Device	Functionality	Dimensions (long -wide -thick)	Weight	Animal model	Reference
Soft optoelectronic system	Deep-brain optogenetics	19 mm × 12 mm × 5 mm	1.4 g	Rat	*(4)*
Flexible PUEH device	DBS for analgesia applications	13.5 mm 9.6 mm × 2.1 mm	0.78 g	Rat	*(5)*
Subdermally implantable platform	Deep-brain optogenetics	13.5 mm × 10.26 mm × 0.89 mm	87 mg	Mice	*(6)*
Our f-BUI device	DBS for epilepsy	13.6 mm × 7.3 mm × 2 mm	0.45 g	Rat	This work

in rodent models.

Supplementary Tab. 2 | Refinement results of KNNS95

Phase	Space group	Fraction	Cell parameters	
rhombohedral (R)	R3m	24.8%	a (Å)	3.9746
			α (deg)	89.8971
orthorhombic (O)	Amm2	40.3%	a (Å)	3.9597
			b (Å)	5.6480
			c (Å)	5.6598
tetragonal (T)	P4mm	34.9%	a (Å)	3.9737
			c (Å)	3.9989
			c/a	1.0218

Supplementary Tab. 3 | Comparison of d_{33}, ε_r, g_{33} and $d_{33} \times g_{33}$ values between D-C, S-C and SP-1-3 composites.

Ceramics/composites	d_{33} (pC N^{-1})	ε_r	g_{33} ($\times 10^{-3}$ V m N^{-1})	$d_{33} \times g_{33}$ ($\times 10^{-15}$ m^2 N^{-1})
D-C	480	2850	19	9120
S-C	420	1500	31.6	13272
SP-1-3	290	534	61.4	17806

Supplementary Tab. 4 | Comparison of the transduction performance of f-BUI and other representative energy harvesters.

Devices	Materials and Structure	Medium	Frequency	Charging power (μw)	Charging rate (μC s^{-1})	Reference
M-gel generator	Two dimensions of $Ti_3C_2T_x$ Mxene	Eco-flex	20 kHz	2.4	2.8	(*7*)
Sm-PUEH	Sm-PMN-PT Single crystal	Water	1 MHz	4.3	6.9	(*5*)
Hybrid-piezoelectrets	FEP and PHA Multilayer	Water	100 kHz	12.9	2.9	(*8*)
U-PEH	KNN-based porous bulk	Water	1 MHz	14.1	21.7	(*9*)
VI-TEG	membrane of perfluoroalkoxy (PFA)	Water	20 kHz	338.4	166	(*10*)
OBHEH	Oscillator	Air	16 Hz	576.7	425	(*11*)
W-PUEH	PZT and polymer; wood structure	Water	40 kHz	865	400	(*12*)
f-BUI	KNN based S-1-3 piezocomposite	Water	1 MHz<br>3 MHz	2465.4	444.1	This work

Supplementary Notes

Supplementary Note 1 | Preparation of KNNS95 ceramics (D-C and S-C)

The lead-free ceramic 0.95(K,Na)(Sb,Nb)O_3-0.05(Bi,Na)ZrO_3-0.2%Fe_2O_3 (KNNS95) powders were synthesized first using a conventional solid-state sintering process, with raw materials including K_2CO_3 (99%), Na_2CO_3 (99.8%), Nb_2O_5 (99.5%), ZrO_2 (99%), Sb_2O_3 (99.99%), Fe_2O_3 (99%) and Bi_2O_3 (99.999%). All the chemicals were mixed after being weighed according to earlier calculations, and then ball milled for 24 h together with ZrO_2 balls and pure alcohol. Then, we broiled the mixtures to make them dry and calcined them at 850 °C for 6 h. A portion of the calcined powders were used to prepare porous ceramics with the burn-out polymer spheres (BURPS) method, using PS microspheres of different diameters and scales as the pore-forming agent. Then the mixtures of powders, PS microspheres and alcohol were ball milled for another 24 h. The calcined powders (with or without PS microspheres) stuck together with 8 wt.% polyvinyl alcohol (PVA) separately, and then they were pressed into shape of two structures (D-C and S-C) under a pressure of 10 MPa. The S-C contain three layers, with dense layer, porous layer, and dense layer in order. After removing the PVA through calcining, the samples were sintered at 1080 °C for 3 h in air. For the follow-up characterization of their electrical properties, we gave them electrodes by pasting silver on both sides and calcining at 600 ℃ for 10 min. Subsequently, all samples were poled in silicone oil under a 2.5 kV mm^{-1} direct current electric field at room temperature for 30 min.

Supplementary Note 2 | Fabrication of dual-transducer

The structural dimension of the dual transducer (1 MHz and 3 MHz) for external transmitter was first designed and optimized based on PZT-4 piezoelectric ceramics with the Krimboltz, Leedom, and Mattaei (KLM) equivalent circuit-based modeling software PiezoCAD. Both the designed 1-MHz and 3-MHz piezoelectric plates have the dimension of a length of 15 mm and a width of 10 mm. The PZT piezoelectric plates with silver electrodes on both surfaces were then fixed into a 3D-printed housing. Two subminiature version A (SMA) connectors were connected with the front and back electrodes using wires and conductive silver paste (E-Solder 3022), respectively. Next, 30 μm-thick parylene-C coating was deposited onto the whole external surface of the two transducers as a protective layer. The two transducers are arranged side by side with a 1 mm spacing between them and are connected using silicone rubber to form a soft connection. Finally, an acoustic matching layer (~1 mm) was created using silicone elastomer (Ecoflex -0030) throughout the front section of the dual transducer for better contact with the skin tissue and acoustic propagation (see **Supplementary Fig. 1** for prepared dual transducer).

Supplementary References

1. Jiang, L. et al. Photoacoustic and piezo-ultrasound hybrid-induced energy transfer for 3D twining wireless multifunctional implants. *Energy Environ. Sci.* **14,** 1490-1505 (2021).
2. Kim, T. et al. Deep brain stimulation by blood-brain-barrier-crossing piezoelectric nanoparticles generating current and nitric oxide under focused ultrasound. *Nat. Biomed. Eng*. **7**, 149-163 (2023).
3. Meng, Y. et al. Applications of focused ultrasound in the brain: from thermoablation to drug delivery. *Nat. Rev. Neurosci*. **17**, 7-22 (2021).
4. Kim, C. Y. et al. Soft subdermal implant capable of wireless battery charging and programmable controls for applications in optogenetics. *Nat. Commun*. **12**, 535 (2021).
5. Zhang, T. et al. Piezoelectric ultrasound energy–harvesting device for deep brain stimulation and analgesia applications. *Sci. Adv.* **8**, eabk0159 (2022).
6. Gutruf, P. et al. Fully implantable optoelectronic systems for battery-free, multimodal operation in neuroscience research. *Nat. Electron*. **1**, 652-660 (2018).
7. Lee, K. H. et al. Ultrasound-Driven Two-Dimensional $Ti_3C_2T_x$ MXene Hydrogel Generator. *ACS Nano* **14**, 3199-3207 (2020).
8. Wan, X. et al. Hybrid‐Piezoelectret Based Highly Efficient Ultrasonic Energy Harvester for Implantable Electronics. *Adv. Funct. Mater.* **32**, 2200589 (2022).
9. Xue, H., Jiang, L., Lu, G. & Wu, J. Multilevel Structure Engineered Lead‐Free Piezoceramics Enabling Breakthrough in Energy Harvesting Performance for Bioelectronics. *Adv. Funct. Mater.* **33**, 2212110 (2022).
10. Hinchet, R. et al. Transcutaneous ultrasound energy harvesting using capacitive triboelectric technology. *Science* **365**, 491-494 (2019).
11. Wang, Z. et al. Broadband omnidirectional piezoelectric–electromagnetic hybrid energy harvester for self-charged environmental and biometric sensing from human motion. *Nano Energy* **113**, 108526 (2023).

12. Hong, Y. et al. A wood-templated unidirectional piezoceramic composite for transmuscular ultrasonic wireless power transfer. *Energy Environ. Sci.* **14**, 6574-6585 (2021).
