# Supplementary material for: Lead-free dual-frequency ultrasound implants for wireless, biphasic deep brain stimulation
Source: Nat Commun. 2024 May 13;15:4017. doi: 10.1038/s41467-024-48250-z (PMC11091107; doi:10.1038/s41467-024-48250-z)
Supplement: Supplementary file 3 — Description of Additional Supplementary Files [file 41467_2024_48250_MOESM3_ESM.pdf]

Description of Additional Supplementary Files

Supplementary Movie 1: Rat moving freely after 30 days of f-BUI implantation
