# Supplementary material for: Lead-free dual-frequency ultrasound implants for wireless, biphasic deep brain stimulation
Source: Nat Commun. 2024 May 13;15:4017. doi: 10.1038/s41467-024-48250-z (PMC11091107; doi:10.1038/s41467-024-48250-z)
Supplement: Supplementary file 5 — Reporting Summary [file 41467_2024_48250_MOESM5_ESM.pdf]

nature portfolio

Corresponding author(s): Jiagang Wu, Laiming Jiang, Hongsong Fan

Last updated by author(s): Jan 8, 2024

Reporting Summary

Nature Portfolio wishes to improve the reproducibility of the work that we publish. This form provides structure for consistency and transparency in reporting. For further information on Nature Portfolio policies, see our Editorial Policies and the Editorial Policy Checklist.

Statistics

For all statistical analyses, confirm that the following items are present in the figure legend, table legend, main text, or Methods section.

n/a	Confirmed	
☐	☒	The exact sample size (*n*) for each experimental group/condition, given as a discrete number and unit of measurement
☐	☒	A statement on whether measurements were taken from distinct samples or whether the same sample was measured repeatedly
☐	☒	The statistical test(s) used AND whether they are one- or two-sided<br>*Only common tests should be described solely by name; describe more complex techniques in the Methods section.*
☐	☒	A description of all covariates tested
☐	☒	A description of any assumptions or corrections, such as tests of normality and adjustment for multiple comparisons
☐	☒	A full description of the statistical parameters including central tendency (e.g. means) or other basic estimates (e.g. regression coefficient) AND variation (e.g. standard deviation) or associated estimates of uncertainty (e.g. confidence intervals)
☐	☒	For null hypothesis testing, the test statistic (e.g. *F*, *t*, *r*) with confidence intervals, effect sizes, degrees of freedom and *P* value noted<br>*Give P values as exact values whenever suitable.*
☒	☐	For Bayesian analysis, information on the choice of priors and Markov chain Monte Carlo settings
☒	☐	For hierarchical and complex designs, identification of the appropriate level for tests and full reporting of outcomes
☒	☐	Estimates of effect sizes (e.g. Cohen's *d*, Pearson's *r*), indicating how they were calculated

Our web collection on statistics for biologists contains articles on many of the points above.

Software and code

Policy information about availability of computer code

Data collection	The crystal structure of the samples was evaluated by the XRD with Cu Kα radiation (Bruker D8 Advanced XRD, Bruker AXS Inc., USA). Reitveld refinement is performed on Findit and MAUD software. Piezoelectric constant d33 was measured by the quasi-static piezo-d33 meter (ZJ-3A, Institute of Acoustics, Chinese Academy of Science). The room-temperature dielectric constant was tested using an LCR analyzer (HP 4980, Agilent, Santa Clara, CA). The temperature-dependence of the dielectric constant at different frequencies was tested by using a dielectric spectrometer (Tonghui 2816A) . The morphology of both surface and cross section was studied by using a scanning electron microscopy (agellan400, FEI Company), where the samples were first polished and then thermally etched. The room temperature and temperature-dependent ferroelectric and strain properties were tested at 1 Hz by using a ferroelectric analyzer (TF 2000, aixACCT Systems GmbH, Germany). Domain structures, local amplitude and phase curves were measured using a piezoelectric force microscopy (PFM, MFP-3D, Asylum Research, Goleta, USA). The generated output voltages of f-BUI were examined by a digital oscilloscope (TBS 2000 Series, Tektronix).The acoustic pressure emitted by the external ultrasonic transmitters was measured at the surface of the implant piezo in the water tank using a hydrophone probe (NH0200, Precision Acoustics Ltd.). FEA simulations were conducted using the COMSOL software (COMSOL Multiphysics 5.3a). Images of cell staining was taken with a confocal laser scanning microscope (LSM 880, Zeiss) using ZEN software (Carl Zeiss Microscopy GmbH, Version 2.3.69.1000). Commercial Fiji ImageJ 1.8.0 was used to perform quantitative analysis of fluorescence intensity. Sections were scanned by an automatic digital slide scanner and analyzed by the Case Viewer 2.1 software (Pannoramic MIDI, 3D HISTECH, Hungary). Micro-CT images were obtained and analyzed using Quantum Image Viewer software (Quantum GX Simple Viewer, PerkinElmer, USA). The motion trace was automatically scored using the OFT-100 opening experiment system (TechMan Software, Sichuan). A Blackrock Neuroport system (Blackrock, USA) was used to measure electrocorticography (ECoG) signals (sampling rate: 30 kHz; bandpass: 1-250 Hz), which were then analyzed using NeuroExplorer software.

Data analysis	Data analysis and plotting was executed with Origin and Graphpad prism

For manuscripts utilizing custom algorithms or software that are central to the research but not yet described in published literature, software must be made available to editors and reviewers. We strongly encourage code deposition in a community repository (e.g. GitHub). See the Nature Portfolio guidelines for submitting code & software for further information.

Data

Policy information about availability of data

All manuscripts must include a data availability statement. This statement should provide the following information, where applicable:

- Accession codes, unique identifiers, or web links for publicly available datasets
- A description of any restrictions on data availability
- For clinical datasets or third party data, please ensure that the statement adheres to our policy

All data needed to evaluate the conclusions in the paper are present in the paper and/or the Supplementary Materials. Additional data related to this paper may be requested from the authors.

Human research participants

Policy information about studies involving human research participants and Sex and Gender in Research.

Reporting on sex and gender	NA
Population characteristics	NA
Recruitment	NA
Ethics oversight	NA

Note that full information on the approval of the study protocol must also be provided in the manuscript.

Field-specific reporting

Please select the one below that is the best fit for your research. If you are not sure, read the appropriate sections before making your selection.

☒ Life sciences ☐ Behavioural & social sciences ☐ Ecological, evolutionary & environmental sciences

For a reference copy of the document with all sections, see nature.com/documents/nr-reporting-summary-flat.pdf

Life sciences study design

All studies must disclose on these points even when the disclosure is negative.

Sample size	For all in vitro studies, at least three random samples were selected for statistical analysis in each experiment based on the effect size and overlap between distributions. For all in vivo studies, at least three random samples were selected for statistical analysis in each experiment. The "n" number in the article represents several definitions, including biologically independent samples, independent experiments and cells examined over "n " independent experiments. Please refer to Author Checklist.
Data exclusions	Data from failed devices were excluded from the analysis.
Replication	All experiments were performed in n independent replicates. The n number is specified in the text and or the figure legends
Randomization	Animals were randomly assigned to treatment groups. All samples were randomly assigned to experimental groups.
Blinding	No formal blinding was used in this study. Because all experiments were performed based on standardized protocols and blinding has no effect on the experiment results. Unbiased experimental procedure and date analysis were carried out as far as possible.

Reporting for specific materials, systems and methods

We require information from authors about some types of materials, experimental systems and methods used in many studies. Here, indicate whether each material, system or method listed is relevant to your study. If you are not sure if a list item applies to your research, read the appropriate section before selecting a response.

Materials & experimental systems

n/a	Involved in the study	
☐	☒	Antibodies
☐	☒	Eukaryotic cell lines
☒	☐	Palaeontology and archaeology
☐	☒	Animals and other organisms
☒	☐	Clinical data
☒	☐	Dual use research of concern

Methods

n/a	Involved in the study	
☒	☐	ChIP-seq
☒	☐	Flow cytometry
☒	☐	MRI-based neuroimaging

Antibodies

Antibodies used

Rabbit anti-TNFα (1:500, ab307164, Abcam); Rabbit anti-IL-6 (1:200, R1412-2, Huabio); Mouse anti-CD68 (1:100, ab125212, Abcam); Alexa 555 goat anti-mouse IgG(1:200, ab150114, Abcam); Alexa 555 goat anti-rabbit IgG(1:500, ab150078, Abcam)

Validation

Positive and negative controls of specific-binding (for each of the florescent labeled targets) were included in each experiment. Specific criteria are used to determine antibody eligibility for each application by the manufacturer. We typically test each antibody in multiple repeated test for the same application. Antibody specificity is verified by a continuous gene knockout (KO)-validation program by the manufacturer. To ensure that different batches of the same antibody produced the same results, the manufacturer also assessed the differences between batches using conformance tests. Antibodies were used at the dilutions recommended by the manufacturer and the statements can be found on the manufactures' websites.

Eukaryotic cell lines

Policy information about cell lines and Sex and Gender in Research

Cell line source(s)

Rat pheochromocytoma 12 (PC12) cells were obtained from Type Culture Collection of the Chinese Academy of Science (Shanghai, China).

Authentication

Authentication of the cells was provided by Type Culture Collection of the Chinese Academy of Science (Shanghai, China).

Mycoplasma contamination

We confirm that all cell lines were negative for mycoplasma contamination.

Commonly misidentified lines
(See ICLAC register)

There was no commonly misidentified lines for our study.

Animals and other research organisms

Policy information about studies involving animals; ARRIVE guidelines recommended for reporting animal research, and Sex and Gender in Research

Laboratory animals

Male Sprague Dawley (SD) rats, aged 6-8 weeks, were provided sterile food and water and kept on a 12-hour light-dark cycle (lights on from 8 AM to 8 PM).

Wild animals

No wild animals were used in the study.

Reporting on sex

The analysis of sex-dependent differences was not pursued in this study. The Laboratory animals were provided by Chengdu Dossy Experimental Animals Co., Ltd.

Field-collected samples

The study did not involve samples collected from that field.

Ethics oversight

All animal experiments were completed in agreement with the Animal Care and Use Committee of Sichuan University (approve number: SYXK (Sichuan): 2019-189).

Note that full information on the approval of the study protocol must also be provided in the manuscript.
